# 10-Hydroxy Decanoic Acid and Zinc Oxide Nanoparticles Retrieve Nrf2/HO-1 and Caspase-3/Bax/Bcl-2 Signaling in Lead-Induced Testicular Toxicity

**DOI:** 10.1007/s12011-024-04374-3

**Published:** 2024-09-30

**Authors:** Adham M. Maher, Ghidaa A. Elsanosy, Doaa A. Ghareeb, Samar S. Elblehi, Samar R. Saleh

**Affiliations:** 1 Bio-Screening and Preclinical Trial Lab, Department of Biochemistry, Faculty of Science, Alexandria University, Alexandria, 21511 Egypt; 2 Pharmaceutical and Fermentation Industries Development Centre (PFIDC), The City of Scientific Research and Technological Applications (SRTA-City), Borg Al‑Arab, Alexandria, Egypt; 3 Research Projects Unit, Pharos University, Alexandria, Egypt; 4 Department of Pathology, Faculty of Veterinary Medicine, Alexandria University, Alexandria, 21944 Egypt

**Keywords:** 10-HDA, Apoptosis, Bax/Bcl-2 ratio, Inflammation, Male infertility, Oxidative stress, ZnO-NPs

## Abstract

There has been a significant increase in human exposure to heavy metals (HMs) over the course of the previous century, primarily due to the extensive industrial processes. Male infertility is a prominent complication associated with lead exposure, wherein lead has the potential to accumulate within the testes, resulting in oxidative stress and inflammation. In addition, 10-hydroxydecanoic acid (10-HDA) is a component found in the secretions of worker bees and possesses the capacity to mitigate oxidative stress and prevent inflammation. Due to their advantageous properties, zinc oxide nanoparticles (ZnO-NPs) possess a wide range of applications in the field of biomedicine. This study aimed to assess the therapeutic effect of 10-HDA and ZnO-NPs on testicular toxicity in rats induced by lead acetate (PbAc). PbAc was administered orally for a period of 3 months. Following that, 10-HDA and/or ZnO-NPs were administrated for 1 month. PbAc deformed seminal analysis, decreased seminal fructose and sex hormonal levels, and resulted in the development of histopathological complications. Additionally, PbAc increased MDA and decreased Nrf2 and HO-1 expression, confirmed by the declined antioxidant defense system. Furthermore, an increase in testicular inflammatory markers and the Bax/Bcl-2 ratio was observed subsequent to the administration of PbAc. The administration of 10-HDA and ZnO-NPs demonstrated significant efficacy in the restoration of semen quality, pituitary/gonadal hormones, antioxidants, and  testicular histoarchitecture. Moreover, 10-HDA and ZnO-NPs decreased testicular inflammatory markers and apoptotic proteins (caspase-3 and Bax expression levels). In conclusion, combining 10-HDA and ZnO-NPs demonstrated synergistic potential in treating PbAc-induced testicular toxicity, thereby presenting a promising approach in nanomedicine and natural drugs.

## Introduction

Impaired fertility has garnered significant attention as a prominent social concern in developed and industrialized countries [1]. In recent years, there has been a significant increase in the number of infertile couples. Infertility affects approximately 8–17% of couples worldwide. Approximately 50% of cases are caused by male infertility [2, 3]. The characteristic features of male infertility include diminished motility, reduced sperm concentration, and abnormal sperm morphology. Numerous etiological factors contribute to male infertility, including diet, environmental pollutants, exposure to free radicals, and drug treatments [4].

Heavy metals (HMs) refer to a set of naturally occurring metalloids or metals with large densities and atomic weights [5]. HMs naturally exist in the environment in considerable amounts. However, the balance of these metals in the water and soil was disrupted due to the processes of industrialization and urbanization [6]. HMs can be classified into two categories: essential metals, such as Zn, Co, Cu, Mn, Mg, Se, Cr, Mo, and Fe, and non-essential metals, such as Hg, Cd, and Pb [7]. Essential metals are a class of micronutrients that play a crucial role in biological systems when present in low concentrations. However, their detrimental effects become apparent when they exceed a specific threshold. In contrast, non-essential metals lack biological value and exhibit toxicity even at minimal concentrations. When the HMs intake rate exceeds the excretion rate, HMs tend to accumulate within tissues [8]. The ability of these elements to accumulate in living organisms and their non-biodegradable properties has demonstrated their potential to induce detrimental effects on specific organs, thereby capturing the interest of researchers in the field of heavy metal toxicity. The extent of heavy metal toxicity is influenced by various factors, including the dose, modality, and duration of exposure, as well as gender, genetics, age, and nutritional status [6]. The detrimental effects of non-essential heavy metals (HMs) stem from their capacity to modify the antioxidant system, generate reactive oxygen species (ROS), interact with and break down DNA, bind to unbound sulfhydryl groups, and displace binding sites on a vast array of enzymes, receptors, and transport proteins. Therefore, a multitude of adverse effects on biological systems are manifested. For instance, disorders such as compromised immune system function, metabolic impairment, imbalanced hormone levels, organ dysfunction, congenital abnormalities, and malignancy were documented [9–11].

Lead (Pb) is widely recognized as one of the most abundant toxic heavy metals found in the environment [12]. Due to the Industrial Revolution, substantial amounts of this metal inhabited the air, water, and soil, causing severe health problems to all living organisms [12]. Current sources of lead toxicity include water pipes, pesticides, batteries, cables, and gasoline additives [13]. The toxicity of lead is attributed to its extended biological half-life, which enables its accumulation in various organs such as the bone, liver, kidneys, brain, and testes. This accumulation results in a diverse array of physiological and biochemical changes, ultimately leading to conditions such as anemia, renal failure, neurological dysfunctions, impaired fertility, and eventually death [6, 14, 15]. Lead can introduce toxicity by increasing the generation of ROS while impairing the antioxidant defense system, causing oxidative stress. Furthermore, lead can disrupt testosterone production and harm cells involved in the development of sperm, such as Sertoli cells, Leydig cells, and Spermatogonia cells, causing infertility and decreased sex drive [12, 16]. Markets are flooded with synthetic products to deal with infertility problems, accompanied by uncertain results and serious side effects. Common contemporary therapeutic interventions encompass antibiotics, antiphlogistics, kallikrein, corticosteroids, and hormone preparations [11, 17, 18].

Natural products have long been regarded as therapeutic agents in the field of folk medicine due to their significant efficacy in promoting human health. Many of these natural products are dispersed in the pharmaceutical market owing to their defensive role against the damaging effects of oxidative stress, inflammation, and drug side effects. Bioactive compounds, whether in isolation or in combination, can be employed as natural products to exert beneficial effects on the male reproductive system [19]. Bee-derived products possess a high concentration of bioactive compounds, rendering them suitable for incorporation into complementary medication, such as treating male reproductive impairment [20]. Royal Jelly (RJ) is produced by nurse bees and secreted from their mandibular and hypopharyngeal glands. In addition, it serves as the sole dietary source for adult queens throughout their lifespan [21–23]. The saturated hydroxyl fatty acid known as 10-hydroxydecanoic acid (10-HDA) is widely recognized as one of the primary fatty acids present in propolis, honey, and RJ [24–29]. The majority of RJ fatty acids are diacids or hydroxyls. The hydroxyl and carboxyl groups located at the head and tail of the carbon chain possess the ability to facilitate the removal of molecular oxygen and the capture of reactive oxygen species that are detrimental to biological processes. These functional groups contribute to the manifestation of biological activities such as antioxidant and anti-inflammatory properties [28]. 10-HDA has a variety of unique physiological activities, including immune-regulating, glucose-regulating activity, anticancer, bactericidal, antioxidant, and anti-inflammatory properties [27, 29–31].

Nanoparticles (NPs) are miniature particles with a size below 100 nm. The significance of particles at the nanoscale was first recognized by scientists when they observed that the size of a substance can influence its properties [32]. Additionally, the modification of the physical, optical, and surface properties of NPs has provided them with a diverse array of potential applications, including their use in medicinal applications for diagnosis, imaging, and therapy [33]. Recently, the beneficial effects of zinc oxide nanoparticles (ZnO-NPs) have facilitated their extensive utilization in various biological and animal scientific contexts due to their outstanding characteristics of solubility, compatibility with biological systems, low price, and minimal toxicity. The topographies of ZnO-NPs allow them to simulate biomolecules that facilitate the regulation of cellular homeostasis through their antioxidant properties [34–36]. Therefore, ZnO-NPs have garnered significant interest across various biomedical fields due to their antidiabetic, antibacterial, anticancer, antioxidant, and anti-inflammatory activities [37]. Furthermore, the detoxifying potential of ZnO-NPs against heavy metal toxicity has been previously examined. Specifically to heavy metals, these particles demonstrated the ability to alleviate allergic dermatitis in rats that were induced with lead oxide [38]. Also, the administration of ZnO-NPs has been shown to mitigate CdCl_2_-induced tongue toxicity in albino rats [39].

The pharmacological activity of 10-HDA, the second most abundant fatty acid in RJ, has been the subject of limited studies, primarily focusing on cellular mechanisms [22]. Therefore, the rationale for conducting this study was based on the scarcity of research regarding the antioxidant and anti-inflammatory characteristics of 10-HDA in alleviating testicular damage resulting from lead exposure, as well as its potential synergistic effects when combined with ZnO-NPs. The primary aim of this study was to assess the efficacy of these interventions in ameliorating lead-induced testicular damage by examining various markers related to antioxidant activity, anti-inflammatory, and anti-apoptotic pathways.

## Material and Methods

### Chemicals

Trichloroacetic acid (TCA, Cat. No. T6399), Tris–HCl (Cat. No. T15760), 5,5′-dithiobis(2-nitrobenzoic acid) (DTNB, Cat. No. D8130), reduced glutathione (GSH, Cat. No. G4251), sulfosalicylic acid dihydrate (Cat. No. S2130), and 10-HDA (99% purity, Cat. No. 379700) were obtained from Sigma-Aldrich, St. Louis, USA. Zinc oxide nanoparticles (ZnO-NPs, 30–50 nm, purity 99.9%, Cat. No. NRL03041) were obtained from Nano Research Lab, Jamshedpur, India. Lead (II) acetate trihydrate (Pb(CH_3_COO)_2_⋅3H_2_O, PbAc) was purchased from Alpha Chemika Co. Andheri, west-Mumbai, India (Cat. No. AL2686). A specific seminal fructose kit was purchased from Spectrum Diagnostics, Egypt (Cat. No. FR2310). GENEzol™ Reagent was purchased from Geneaid, Taiwan (Cat. No. GZR100). TOPscript™ RT DryMIX (dT18/dN6) and TOPreal™ qP CR 2X PreMIX (SYBR Green with low ROX) were obtained from Enzynomics, Korea (Cat. Nos. RT220 and RT500M, respectively). Primers were obtained from Willowfort, UK. Roche Elecsys testosterone II (Cat. No. 05200067190), Roche Elecsys luteinizing hormone (LH, Cat. No. 11732234122), and Roche Elecsys follicle-stimulating hormone (FSH, Cat. No. 11775863122) kits were purchased from Roche Diagnostics, Indianapolis, IN. All chemicals used were of high analytical degree.

### Characterization of Zinc Oxide Nanoparticles

Zinc oxide powder was suspended in 10 ml of ethanol and left for 1 h in an ultrasonic cleaner [40]. Subsequently, a copper grid coated with a Formvar membrane was loaded with 4 µl of the ZnO-NPs sample [41]. The grid was subsequently put inside the JEOL-JSM-1400 PLUS (TEM) transmission electron microscope’s sample holder, and the sample was examined [42].

Scanning Electron Microscopy (SEM) (JEOL JSM-IT 200) equipment was used to determine the optical properties and nanostructure morphology of ZnO-NPs. Therefore, the solid was coated with a thin conductive gold coating on a double-sided carbon tape, followed by subsequent analysis [43]. The element composition of the samples and the purity of ZnO-NPs were determined using energy-dispersive X-ray spectroscopy (EDX) (JOEL-IT 200) [41, 44].

### Animals and Experimental Design

**Fig. 1:**
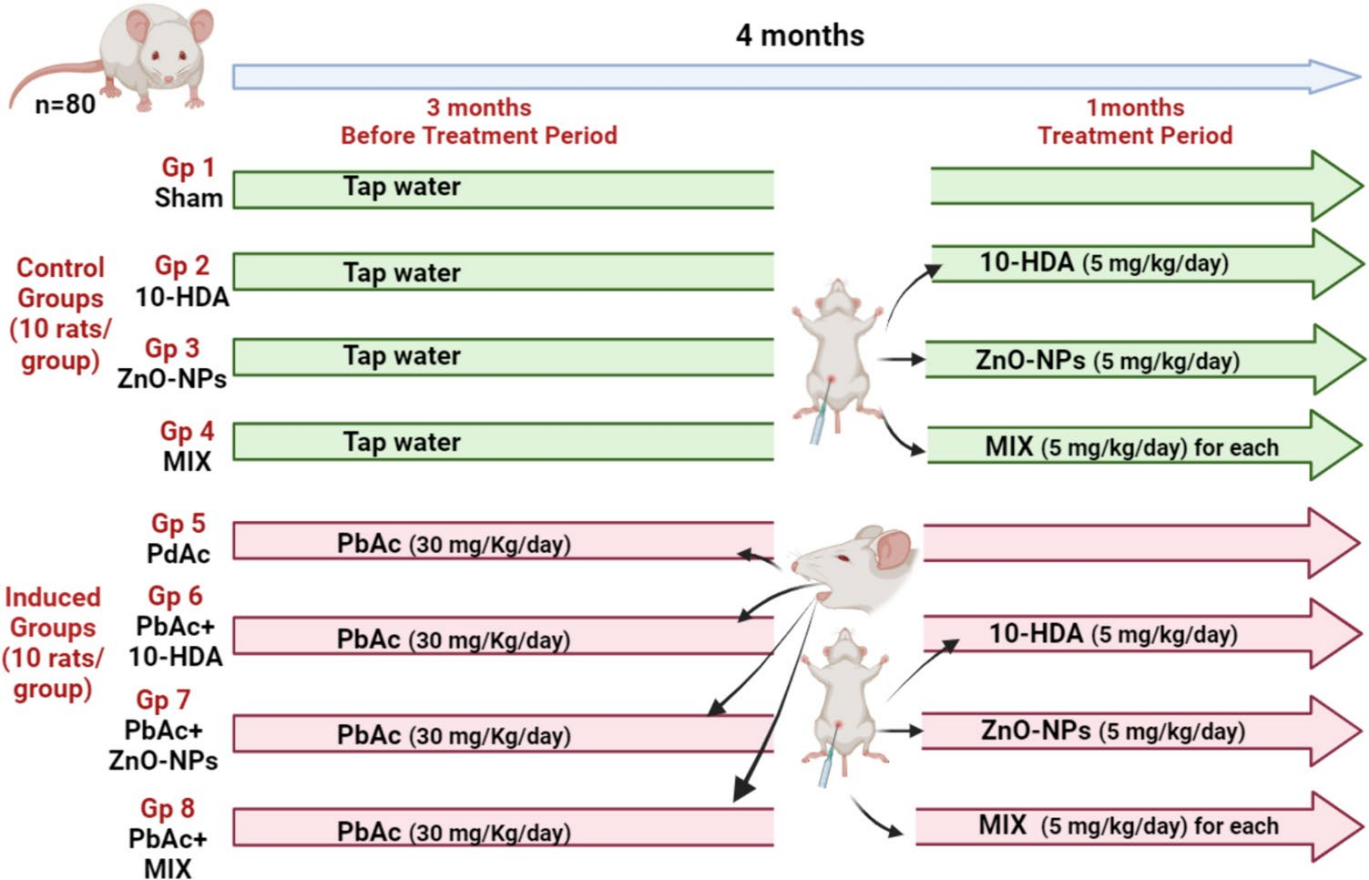
Experimental design and group classification

The present study utilized a sample of 80 mature male Sprague Dawley rats weighing between 150 and 200 g and aged 10–12 weeks. The animals were obtained from the Institute of Graduate Studies and Research’s animal house at Alexandria University, Egypt. All animal procedures adhered to the ARRIVE guidelines. All animal treatments were conducted in accordance with the ethical guidelines for scientific research established by the Institutional Animal Care and Use Committee (IACUC) of Alexandria University (AU: 04 22 10 22 1 01). The animals were housed in polypropylene cages (5 rats/cage) in a well-ventilated room in a regulated setting for a 12:12-h diurnal period. Rats had unrestricted access to food (growing fodder 21% protein, Elfagr Company, Egypt) and water. Following a week of acclimation, rats were randomly divided into eight groups (10 animals/group), as shown in Fig. 1. The control groups included (1) sham group, (2) 10-HDA group, (3) ZnO-NPs group, (4) mix group (mixture = 10-HAD + ZnO-NPs). The infertility-induced groups included (1) PbAc group, (2) PbAc + 10-HDA group, (3) PbAc + ZnO-NPs group, and (4) PbAc + mix group (mixture = 10-HAD + ZnO-NPs). Lead-induced infertility was achieved using oral gavage of PbAc (30 mg/kg/day) for 3 months [45, 46]. Doses were calculated and prepared weekly in correspondence with the body weight of each rat. Pb-acetate was formulated via dissolution in distilled water. Following the Pb-acetate induction, a treatment scheme was initiated by the administration of 10-HDA (5 mg/kg body weight) [47] and/or ZnO-NPs (5 mg/kg body weight) [48–50]. The doses were prepared by dissolving the corresponding doses in Polly ethylene glycol, taking into consideration the body weight of each rat. Treatments were administered intraperitoneally on a daily basis at a consistent time each day for a duration of 4 weeks.

### Collecting and Handling Blood and Testicular Tissues

Following the conclusion of the experimental period, the rats were fasted overnight and then were anesthetized with isoflurane inhalation (4% for 2–3 min) in preparation for decapitation [51]. Blood samples were collected from the inferior vena cava, followed by centrifugation to obtain the sera, and stored at − 20 °C. For biochemical and molecular evaluations, the left testes were stored at a temperature of − 80 °C. The testicular tissue was homogenized in Tris HCl buffer (20 mM, pH 7.4) at a 1:9 (w/v) ratio. This resulted in the formation of a supernatant, which was subsequently stored at − 80 °C for the purpose of analyzing fructose levels, inflammation markers, apoptotic markers, and biomarkers associated with oxidative stress. Histopathological examination was conducted on the right testicles of each animal using 10% neutral formalin fixation. The epididymis was isolated for seminal analysis.

### Epididymis Seminal Quality and Seminal Fructose Level

The epididymis was carefully divided using scissors to release the spermatozoa into a clean petri dish. The dish contained 2 ml of Ham’s F-10 and 0.5% bovine serum albumin (BSA). A comprehensive assessment of the epididymis sperm count, morphological index, and motility was conducted using a hemocytometer. All equipment and materials were preheated and kept at 37 °C. In order to ensure accuracy, four counts were conducted on each sample, and the average was calculated [52, 53].

Seminal fructose level was measured using the spectrophotometric method according to a previous study [54]. A homogeneous mixture consisting of 500 µl TCA (1 mol/l) combined with either standard fructose (300 mg/dl), supernatant of testis homogenate, or distilled water (blank) was prepared. After 10 min at ambient temperature, the mixture was centrifuged for 10 min at 3000 rpm. Subsequently, 1 ml of hydrochloric acid (9 mol/l) and 50 μl of resorcinol (9 mmol/l) were combined with the supernatant, mixed, and then set for 5 min in a bath of boiling water. The produced color was measured in relation to the blank at 495 nm. The analysis of fructose levels in testicular tissue was conducted and expressed as mg/g.

### Determination of Testicular Lead Content

Wet digestion was utilized to determine the Pb content in the testes. First, 300 mg of the testes tissue was dehydrated at 120 °C overnight in an oven. Then, the dried sample was carefully placed in a cold muffle furnace and subjected to a gradual temperature increase. The temperature rose from ambient to 450 °C at a constant rate of 50 °C per hour. Subsequently, the sample was subjected to digestion using concentrated nitric acid and further dried in the muffle at 450 °C for 1 h. The resulting powder was dissolved in 1 N hydrochloric acid and then appropriately diluted using 0.2% nitric acid. Lead concentration in the testicular sample was detected utilizing flame atomic absorption spectrophotometry, specifically employing the Shimadzu model (AA-6650). The absorbance was measured at 283.3 nm, following the protocol defined by Szkoda and Zmudzki [55].

### Serum Pituitary/Gonadal Hormones

The measurement of serum luteinizing hormone (LH), follicle-stimulating hormone (FSH), and testosterone levels was conducted in accordance with the manufacturer’s instructions of the ELISA kits (COBAS, Indianapolis, IN 46256, USA). An ELISA reader (ELx800, Bio-TEK, Winooski, VT, USA) was used to measure the absorbance at 450 nm. LH and FSH levels were reported as µU/ml, whereas testosterone was expressed as ng/dl.

### Testicular Oxidative Stress Markers

The frozen testicular tissues were homogenized in a solution containing a protease inhibitor and lysis buffer (1% Triton X-100, 150 mM NaCl, 10 mM Tris, pH 7.4), then centrifuged for 10 min at 4 °C at 10,000 g. Following that, the supernatant was collected. Malondialdehyde (MDA) level was assessed as a marker of lipid peroxidation. Under acidic conditions, MDA combines with thiobarbituric acid (TBA) to generate a pink product that, when heated, can be detected spectrophotometrically at 532 nm. The amount of MDA in the testicles was reported as µmol/mg protein [56]. Testicular nitric oxide (NO) content was measured spectrophotometrically following the method by Montogomery and Dymock [57]. The application of a diazotizing agent, such as sulfanilamide, in an acidic environment to nitrite resulted in the formation of an intermediate diazonium salt. This salt subsequently reacts with N-naphthyl-ethylenediamine, forming a stable azo compound. The resulting deep purple hue was measured at 540 nm and stated as mM/mg protein. Reduced glutathione (GSH) was measured using Ellman’s method [58]. GSH reacts with 5,5′-dithio-bis-2-nitrobenzoic acid (DTNB), creating a 2-nitro-5-thiobenzoic acid product. The yellow-colored product was detected at 412 nm and represented as µmol/mg protein [59]. The assessment of glutathione-S-transferase (GST) activities in the testicles was conducted following the protocol outlined by Habig [60]. GST catalyzes the reaction between GSH and p-nitrobenzyl chloride, forming glutathione nitrobenzyl, which was detected at 310 nm and quantified as U/mg protein. For superoxide dismutase (SOD), the inhibitory effect of SOD on the pyrogallol autooxidation was assessed spectrophotometrically at 420 nm. The amount of enzyme required to decrease pyrogallol autooxidation by 50% is equivalent to one unit of SOD activity. The SOD activity was reported as U/mg protein [61].

For the estimation of enzymes’ specific activity (U/mg protein), the total testicular protein was measured, with BSA (1 mg/ml) serving as a reference in accordance with the Lowry method [62].

### Quantitative Real-Time Reverse Transcription PCR Analysis (qRT-PCR)

The extraction of total RNA (50–100 mg) from testicular tissue was conducted in accordance with the manufacturer’s instructions, utilizing GENEzol™ Reagent. For estimating the concentration and purity of the isolated RNA, a NanoDrop 2000 spectrophotometer (Thermo Scientific, USA) was used to measure the absorbance at 260 nm and 280 nm (A260/280). Samples with A260/280 ≥ 1.8 were considered for further analysis. Using TOPscriptTM RT DryMIX (dT18/dN6) and 5 μg of the extracted RNA, cDNA synthesis was carried out. For qRT-PCR analysis, 1 μl cDNA was mixed with 1 µl forward primer, 1 µl reverse primer, 10 μl TOPreal™ qPCR 2X PreMIX (SYBR Green with low ROX), and RNase-free water to reach a final volume of 20 μl per well. To perform quantitative PCR, the CFX96TM Real-Time System was utilized (BIO-RAD, USA) with the following thermal protocol: initial denaturation lasting 12 min at 95 °C, then 45 cycles of denaturation for 10 s at 95 °C, annealing for 30 s at 52 °C, and extension for 30 s at 72 °C. Duplicate samples were loaded for each analysis. The comparative 2^−ΔΔCT^ method was exploited to determine the fold change in the target gene compared to the sham control calibrator. The GAPDH mRNA level was used to normalize the data. The targeted genes’ primer sequences for glyceraldehyde phosphate dehydrogenase (GAPDH, NM_017008.4), tumor necrosis factor-alpha (TNF-α, NM_012675.3), interleukin-1β (IL-1β, NM_031512.2), IL-6 (NM_031168.1), IL-8 (NM_017183.2), B-cell lymphoma 2 (Bcl-2, NM_016993.2), Bcl-2 Associated X-protein (Bax, NM_017059.2), and cysteine–aspartic acid protease (caspase-3, NM_012922.2) are adopted from [63], as depicted in Table 1.

**Table 1:** Primer sequences of genes analyzed in real-time PCR

Gene name	Primer sequence	
GAPDH	F 5′-AGATCCACAACGGATACATT- 3′	R 5′-TCCCTCAAGATTGTCAGCAA- 3′
TNF-α	F 5′-ACACACGAGACGCTGAAGTA- 3′	R 5′-GGAACAGTCTGGGAAGCTCT- 3′
IL-1* β*	F 5′-GACTTCACCATGGAACCCGT- 3′	R 5′-GGAGACTGCCCATTCTCGAC- 3′
IL-6	F 5′-GCCAGAGTCATTCAGAGCAATA -3′	R 5′-GTTGGATGGTCTTGGTCCTTAG-3′
IL-8	F 5′-CATTAATATTTAACGATGTGGATGCG-3′	R 5′-GCCTACCATCTTTAAACTGCACAAT-3′
Bcl-2	F 5′-GCAGCTTCTTTCCCCGGAAGGA-3′	R 5′-AGGTGCAGCTGACTGGACATCT-3′
Bax	F 5′-AACTTCAACTGGGGCCGCGTGGTT-3′	R 5′-CATCTTCTTCCAGATGGTGAGCGAG-3′
Caspase-3	F 5′-GTGGAACTGACGATGATATGGC-3′	R 5′-CGCAAAGTGACTGGATGAACC-3′

*GAPDH*, glyceraldehyde-3-phosphate dehydrogenase; *TNF-α*, tumor necrosis factor-alpha; *IL-1 β*, interleukin-1beta; *IL-6*, interleukin-6; *IL-8*, interleukin-8; *Bcl-2*, B-cell lymphoma 2; *Bax*, Bcl-2 Associated X-protein; *Caspase-3*, Caspase 3

### Western Blotting Analysis

Testes tissue protein extract was used to examine the levels of protein expression for nuclear factor erythroid 2–related factor-2 (Nrf2), heme oxygenase-1 (HO-1), and inhibitor of nuclear factor κβ kinase (Phospho-IKK α/β). This was done following the established protocol outlined by Mahmood and Yang (2012). Specific antibodies were utilized, including rabbit monoclonal antibodies for HO-1, Phospho-IKK α/*β*, and Nrf2 (designated as #2697 s, #82206 s, and #20733, respectively, from Cell Signaling Technology, USA). Additionally, a rabbit monoclonal antibody β-actin 13E5 (#4970, Cell Signaling Technology, USA) and goat anti-rabbit IgG, AP-linked Ab (#7054, Cell Signaling Technology, USA) were utilized. In order to visualize the protein bands, NBT/BCIP solution (Thermo Fisher Scientific, USA) was utilized, obeying the instructions provided by the manufacturer. The protein bands’ intensity was quantified using Quantity One software (Bio-Rad Laboratories, USA). For accurate comparison, the intensity of the protein bands was normalized to β-actin. The results were then reported as a relative intensity normalized to β-actin, facilitating interpretation and analysis.

### Combination Index Analysis

The quantitative assessment of the combined effects of ZnO-NPs and 10-HDA was done by calculating the combination index (CI). The combination therapy has the potential to yield synergistic (when CI < 1), antagonistic (when CI > 1), or additive (when CI = 1) outcomes compared to using each treatment separately. The determination of the combination index was conducted following the methodology described by Zhou [64] and Saleh [2]. For the computation of the combination index, the predictive value for the combination of 10-HDA/ZnO-NPs was initially determined using the following equation:

$$\text{The Predicted value}=\left(\frac{\text{Observed value for }10-\text{HDA }}{\text{Control value }}+\frac{\text{Observed value for ZnO}-\text{NPs }}{\text{Control value}}\right) \times \text{ Control value}$$

The CI was calculated using the formula:

$$\text{CI}=\frac{\text{Observed value }}{\text{Predicted value of the combination}}$$

### Testicular Histopathological Examination

Testicular tissue specimens were immediately preserved in phosphate-buffered formalin (10%, pH 7.4) after necropsy and left for at least 24 h prior to undergoing treatment using the standard paraffin embedding method [65]. Five micrometer–thick sections were cut, placed on xylene-deparaffinized slides, and rehydrated with ethanol at progressively decreasing concentrations. Hematoxylin and eosin (H&E) staining was applied to slides for routine histopathological settings. A Leica DM500 light microscope was used for a blind analysis of stained sections, and a digital camera was used to take pictures of the sections at a magnification of 100 (EC3, Leica, Germany). A histological lesion-scoring system was used to indicate the severity of histopathological lesions. Within each animal group, a total of ten slides stained with H&E were examined. Each slide was assigned to a specific rat, and the pathological lesions were assessed using a blind method. The grading process involved evaluating ten random fields per slide, which included degenerated germinal epithelial cells, buckled basement membrane, sloughed degenerated germinal epithelial cells, intertubular vascular congestion, and interstitial edema. The intensity of pathological lesions was determined by assessing the percentage of damaged tissue across the entire section. Based on the percentage of damaged tissue throughout the entire section, the intensity of pathological lesions was expressed as None (0): normal histology with no impact from the field under investigation, Mild (1): 5–25% of the scanned field was implicated, Moderate (2): 26–50% of the field under investigation was affected, Severe (3): 50% engagement in the field under examination. In order to determine the overall tissue damage scores, the scores for each parameter were aggregated [66].

### Statistical Analysis

The SPSS software, version 16.0 (Chicago, IL, USA), was used for statistical tests, and the results were expressed as the mean ± SD. Using the LSD test and one-way ANOVA, differences within groups were statistically analyzed. In addition, a *p*-value of < 0.05 was considered statistically significant.

## Results

### Characterization of ZnO-NPs

**Fig. 2:**
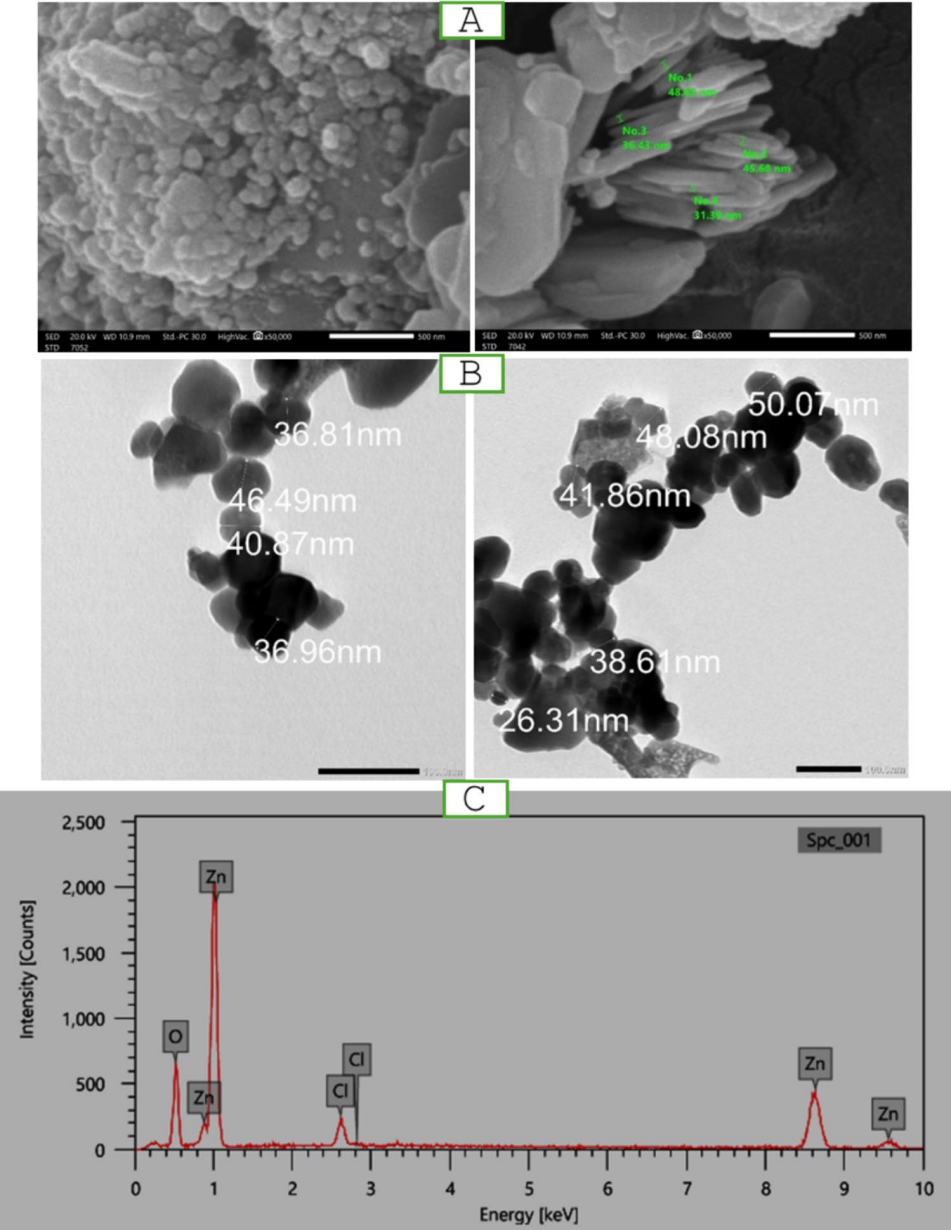
Characterization of ZnO-NPs. (A) SEM micrograph of ZnO-NPs in scale bar 500 nm. (B) TEM micrograph of ZnO-NPs. (C) EDX data of ZnO-NPs

The SEM micrograph of ZnO-NPs shows a spherical homogeneous shape of the nanoparticles, whereas the average particle size for the nanoparticle is about 50 nm (Fig. 2A). The microscopic image from TEM revealed a hexagonal shape for ZnO-NPs (Fig. 2B). The EDX data revealed the presence of two elements: Zn (70.08%) and O (24.60%) and a neglectable amount of Cl (5.31%), thus confirming the high purity of ZnO-NPs, as depicted in (Fig. 2C).

### The Impact of Lead Acetate Exposure and Different Treatments on Seminal Analysis and Seminal Fructose Level Among the Investigated Groups

**Fig. 3:**
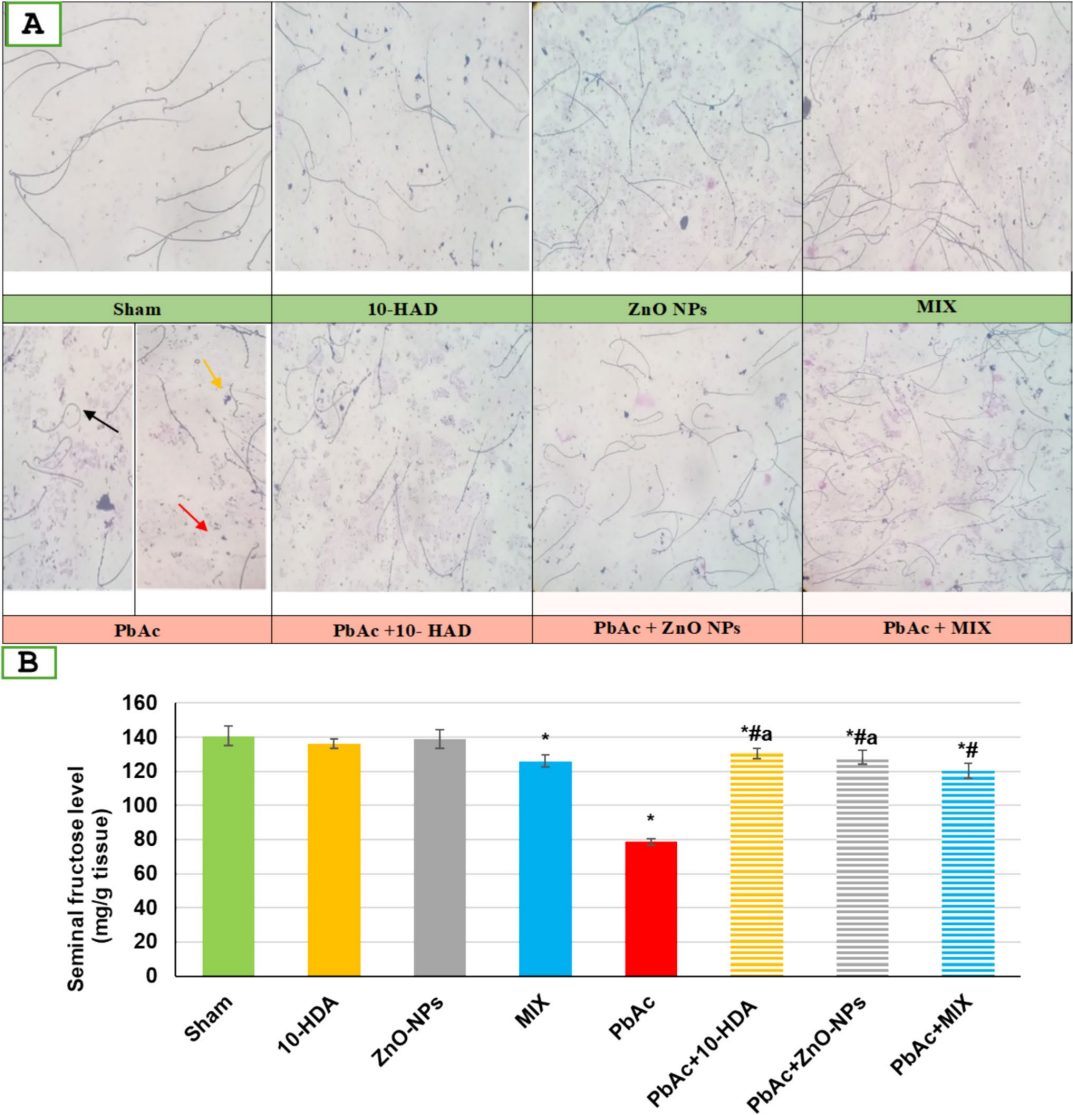
The impact of lead acetate exposure and different treatments on seminal analysis (A) and fructose level (B) in different experimental groups. Sperm morphology was stained using the Diff-Quick procedure. Sham and treated controls (10-HDA, ZnO-NP, and MIX groups) show normal sperm morphology of heads and tails. The pbAc-induced group exhibited sperm abnormalities as coiled tail (black arrow), detached head (red arrow), and detached tail (yellow arrow). PbAc + 10-HDA, PbAc + ZnO-NP, and PbAc + MIX treatments resulted in the restoration of normal sperm morphology (A)

Figure 3A shows normal sperm morphology of sham, 10-HDA, ZnO-NPs, and MIX groups. Conversely, animals exposed to PbAc exhibited anomalous sperm morphology characterized by head and tail abnormalities, specifically round heads and coiled tails. Additionally, detachments were observed in these animals. In contrast, the application of 10-HDA and/or ZnO-NPs treatments resulted in enhanced sperm morphology. Table 2 illustrates that the group exposed to PbAc exhibited a statistically significant decrease (*p* < 0.05) in the number of sperm in the epididymis, as well as a reduction in the morphology index, sperm progressive motility, and percentage of motility during the first, second, and third hour. Furthermore, there was a notable increase in non-progressive motility compared to the control group. In contrast, the lead-induced rats receiving treatments revealed significant (*p* < 0.05) recovered semen quality as related to the PbAc untreated group. Regarding seminal fructose levels (Fig. 3B), PbAc-induced infertility rats showed a significant (*p* < 0.05) decline in seminal fructose levels compared to the sham group. However, administering 10-HDA and/or ZnO-NPs to PbAc-exposed rats resulted in a significant increase (*p* < 0.05) in seminal fructose levels paralleled with infertile rats. The control groups treated with 10-HAD and ZnO-NPs exhibited no significant alterations in seminal fructose levels compared to sham control. In contrast, the control group of rats that were administered the MIX (10-HAD and ZnO-NPs) exhibited a statistically significant reduction (*p* < 0.05) in seminal fructose levels when compared to the sham control group (Fig. 3B).

**Table 2:** The impact of lead acetate exposure and different treatments on seminal analysis including sperm count, morphology index, motility type, and motility time

Parameters		Sham	10-HAD	ZnO-NPs	MIX	PbAc	PbAc + 10-HDA	PbAc + ZnO-NPs	PbAc + MIX
Sperm count (10^6^/ml)		206 ± 4.55	192.8 ± 1.71*	194.75 ± 0.96*	203.5 ± 4 0.65	152.25 ± 5.74*	168.5 ± 1.29*^#a^	191 ± 0.58*^#a^	198.5 ± 1.83*^#^
Morphology index (%)		80.5 ± 1.29	78.8 ± 4.11	79.25 ± 0.96	82.5 ± 1.29	72.5 ± 1.29*	77.5 ± 1.29*^#^	80.0 ± 0.82^#^	79.5 ± 1.29^#^
Motility type (%)	Progressive	59.75 ± 1.71	57.5 ± 1.29	58.5 ± 1.29	56.5 ± 2.08*	49.25 ± 1.71*	52.0 ± 2.16*^a^	53.5 ± 1.29*	55.5 ± 0.58*
	Non-progressive	10.75 ± 2.63	11.5 ± 1.29	11.5 ± 1.29	9.75 ± 0.96	21.5 ± 2.38*	10.0 ± 2.16^#a^	8.5 ± 2.38^#^	8.5 ± 1.29^#^
Motility (%)	1st h	62.25 ± 1.71	64.5 ± 1.29	57.5 ± 1.29*	69.5 ± 1.29*	49.5 ± 1.29*	64.0 ± 2.94^#a^	60.5 ± 1.29^#a^	71.25 ± 2.22*^#^
	2nd h	40.5 ± 1.29	38 ± 0.82*	34.5 ± 1.29*	43 ± 2.16*	28.5 ± 1.29*	39.8 ± 1.71^#a^	35.8 ± 2.75*^#a^	46.5 ± 1.29*^#^
	3rd h	11.25 ± 1.71	9.5 ± 2.38	8.5 ± 1.29	14.8 ± 1.71*	6.75 ± 0.96*	10.0 ± 1.83^#^	7.0 ± 0.82*^a^	13.25 ± 3.30^#^

Values denote the mean ± SD of ten rats/ group. *p ≤ 0.05 vs. control, #*p* ≤ 0.05 vs. PbAc-induced group, and ^a^*p* < 0.05 vs. PbAc + MIX group using ANOVA (one-way)

### The Impact of Lead Acetate Exposure and Different Treatments on Testicular Lead Content Among the Investigated Groups

**Fig. 4:**
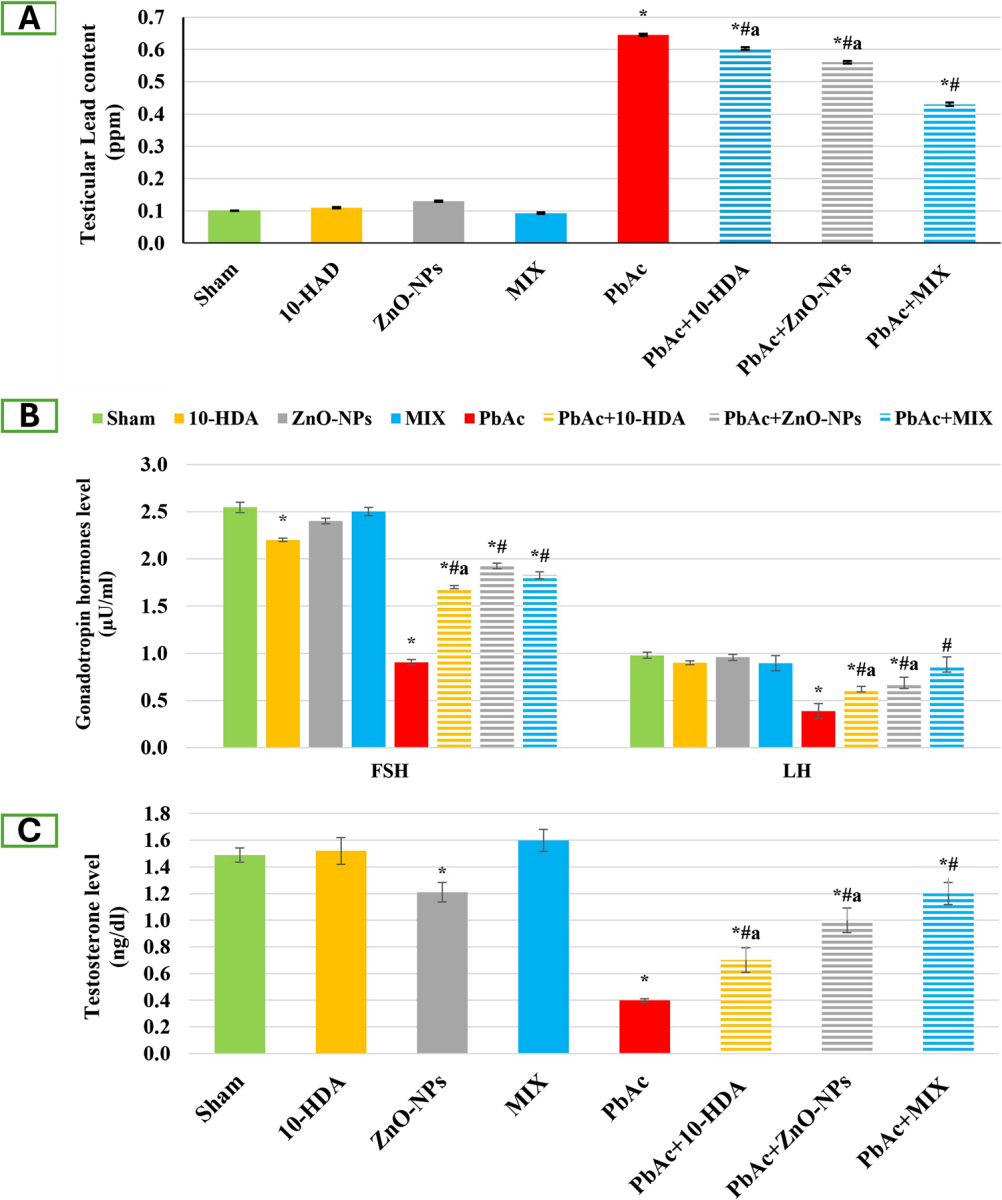
The impact of lead acetate exposure and different treatments on testicular lead content (A) and serum pituitary/gonadal hormones, including follicle-stimulating hormone (FSH) and luteinizing hormone (LH) (B), and testosterone (C). Data are presented as mean ± standard deviation (*n* = 10). *: significant (*p* < 0.05) compared to the sham group, ^#^: significant (*p* < 0.05) compared to the PbAc-induced group, ^a^: significant (*p* < 0.05) compared to the PbAc + MIX group. 10-HDA, 10-hydroxydecanoic acid (5 mg/kg/day); ZnO-NPs, zinc oxide nanoparticles (5 mg/kg/day); MIX, mixture of 10-hydroxydecanoic acid + zinc oxide nanoparticles; PbAc, lead acetate (30 mg/kg/day); PbAc + 10-HDA, lead acetate + 10-hydroxydecanoic acid; PbAc + ZnO-NPs, lead acetate + zinc oxide nanoparticles; PbAc + MIX, lead acetate + mixture of 10-hydroxydecanoic acid + zinc oxide nanoparticles

Figure 4A shows variations in testicular lead levels among the various experimental groups. The PbAc-induced group exhibited a statistically significant increase (*p* < 0.05) in testicular lead content when compared to the sham group. The administration of 10-HDA and/or ZnO-NPs to PbAc-toxified rats resulted in significant (*p* < 0.05) reductions in testicular lead levels, with a more pronounced reduction observed in the MIX treatment compared to PbAc-induced rats. Furthermore, the rats that received treatment exhibited a statistically significant increase (*p* < 0.05) in comparison to the rats in the sham group. No statistically significant differences in testicular lead levels were observed in the control-treated groups (10-HDA and/or ZnO-NPs) compared to the sham group.

### The Impact of Lead Acetate Exposure and Different Treatments on Pituitary/Gonadal Hormones Among the Investigated Groups

The study revealed a significant decrease (*p* < 0.05) in serum levels of FSH, LH, and testosterone in the PbAc-testicular intoxified group compared to the sham group (Fig. 4B and C). Nevertheless, 10-HDA and/or ZnO-NPs treatments caused a significant elevation (*p* < 0.05) in the levels of these hormones compared with PbAc-intoxified rats. Regarding healthy rats receiving the single or combined treatments, a significant (*p* < 0.05) reduction in serum FSH level was associated with 10-HDA treatment. In contrast, ZnO-NP administration caused a significant decline (*p* < 0.05) in serum testosterone levels compared to the sham group. However, the MIX therapy did not cause any significant changes in the serum levels of FSH, LH, and testosterone compared to sham rats (Fig. 4B and C).

### The Impact of Lead Acetate Exposure and Different Treatments on Testicular Antioxidant and Lipid Peroxidation Biomarkers Among the Investigated Groups

Comparative analysis revealed notable changes in the oxidative stress indices between rats treated with PbAc and the sham rats. The testicular activity of superoxide dismutase (SOD) and glutathione (GST), as well as the GSH content, exhibited a statistically significant decrease (*p* < 0.05) (Table 3). Conversely, the oxidative stress markers exhibited a significant improvement (*p* < 0.05) following the administration of 10-HDA and/or ZnO-NPs treatments. No significant alterations in the antioxidant markers were observed in the majority of control rats that received treatments. Furthermore, it was observed that rats exposed to PbAc exhibited a statistically significant increase (*p* < 0.05) in the lipid peroxidation indicator (MDA) when compared to the control group. In contrast, the administration of either the single or mixed treatments resulted in a statistically significant decrease (*p* < 0.05) in MDA levels in rats that received PbAc. Alternatively, administration of the single or mixed treatments caused a significant decline (*p* < 0.05) in MDA levels in rats receiving PbAc, where the mixed treatment exhibited greater efficacy. Single or combined-treated control rats did not display any significant change in MDA level compared to the sham rats (Table 3).

**Table 3:** The impact of lead acetate exposure and different treatments on testicular antioxidant and lipid peroxidation biomarkers

Parameters	Sham	10-HDA	ZnO-NPs	MIX	PbAc	PbAc + 10-HDA	PbAc + ZnO-NPs	PbAc + MIX
SOD (U/mg protein)	266.48 ± 0.51	262.65 ± 1.29	251.00 ± 0.70*	287.10 ± 0.97*	141.74 ± 0.30*	236.83 ± 1.31*^#a^	242.80 ± 0.21*^#a^	260.20 ± 2.20^#^
GST (U/mg protein)	740.60 ± 27.85	671.00 ± 15.87*	735.60 ± 11.93	644.10 ± 32.63*	454.11 ± 26.84*	629.91 ± 24.54*^#^	570.00 ± 28.60*^#a^	624.60 ± 21.21*^#^
GSH (mM/mg protein)	3.55 ± 0.13	3.93 ± 0.36	3.78 ± 0.17	3.70 ± 0.22	1.90 ± 0.29*	2.85 ± 0.13*^#a^	3.63 ± 0.78^#^	3.43 ± 0.42^#^
MDA (mM/mg protein)	0.16 ± 0.05	0.19 ± 0.04	0.18 ± 0.02	0.16 ± 0.01	0.23 ± 0.01*	0.21 ± 0.04	0.21 ± 0.04	0.16 ± 0.03^#^

Data correspond to the mean ± SD of ten rats/ group. *p ≤ 0.05 vs. control, #*p* ≤ 0.05 vs. PbAc-induced group, and ^a^*p* < 0.05 vs. PbAc + MIX group using ANOVA (one-way), *n* = 10

**Fig. 5:**
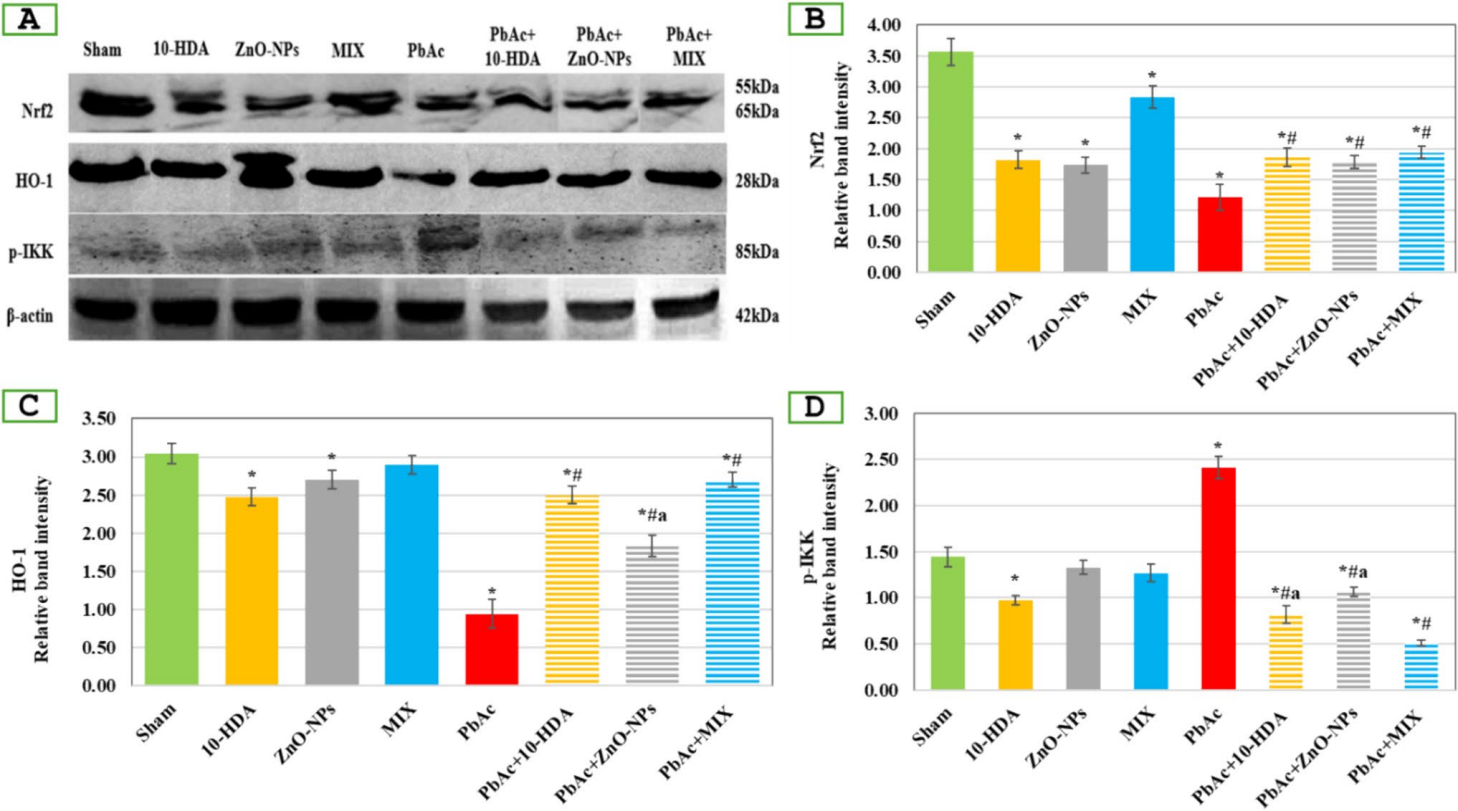
Western blot analysis of testicular nuclear factor erythroid 2–related factor 2 (Nrf2), heme oxygenase-1 (HO-1), and phosphorylated inhibitor of nuclear factor κβ kinase (p-IKK) in the different experimental groups. (A) The western immunoblots. (B–D) Relative intensity of the protein bands analyzed by densitometry. β-actin was probed as an internal control for relative intensity analysis. Data is shown as mean ± standard deviation (*n* = 3). *: significant (*p* < 0.05) compared to the sham group, ^#^: significant (*p* < 0.05) compared to the PbAc-induced group, ^a^: significant (*p* < 0.05) compared to the PbAc + MIX group. 10-HDA, 10-hydroxydecanoic acid (5 mg/kg/day); ZnO-NPs, zinc oxide nanoparticles (5 mg/kg/day); MIX, mixture of 10-HDA + ZnO-NPs; PbAc, lead acetate (30 mg/kg/day); PbAc + 10-HDA, lead acetate + 10-hydroxydecanoic acid; PbAc + ZnO-NPs, lead acetate + zinc oxide nanoparticles; PbAc + MIX, lead acetate + mixture of 10-HDA + ZnO-NPs

Figure 5A–C shows changes in testicular protein content of Nrf2 and HO-1 associated with the various treatments. The PbAc-exposed group exhibited a significant decrease (*p* < 0.05) in Nrf2 and HO-1 protein levels compared to healthy animals. The different treatment regimens (10-HDA and/or ZnO-NPs) resulted in a significant elevation (*p* < 0.05) in Nrf2 and HO-1 protein levels compared to rats exposed to PbAc only. The combined treatment demonstrated greater efficacy in increasing the expression levels of Nrf2 and HO-1. Nonetheless, the protein content of Nrf2 and HO-1 after these treatments remained significantly lower (*p* < 0.05) compared to sham rats. While individual administrations of 10-HDA or ZnO-NPs to healthy rats resulted in statistically significant reductions (*p* < 0.05) in Nrf2 and HO-1 expression levels compared to sham rats, the combined treatment did not yield significant effects (Fig. 5A–C).

### The Impact of Lead Acetate Exposure and Different Treatments on Testicular Inflammatory Markers Among the Investigated Groups

Figure 5D displays the levels of testicular p-IKK protein for each of the designed groups. In comparison to the sham rats, a statistically significant increase (*p* < 0.05) in the expression level of p-IKK protein was observed in rats receiving PbAc. Nevertheless, the observed increase was notably reduced (*p* < 0.05) by the various intervention protocols (10-HDA and/or ZnO-NPs). Furthermore, the administration of 10-HDA to healthy rats resulted in a statistically significant (*p* < 0.05) reduction in the expression level of p-IKK. In contrast, the other control groups did not exhibit any significant differences when compared to the sham control.

**Fig. 6:**
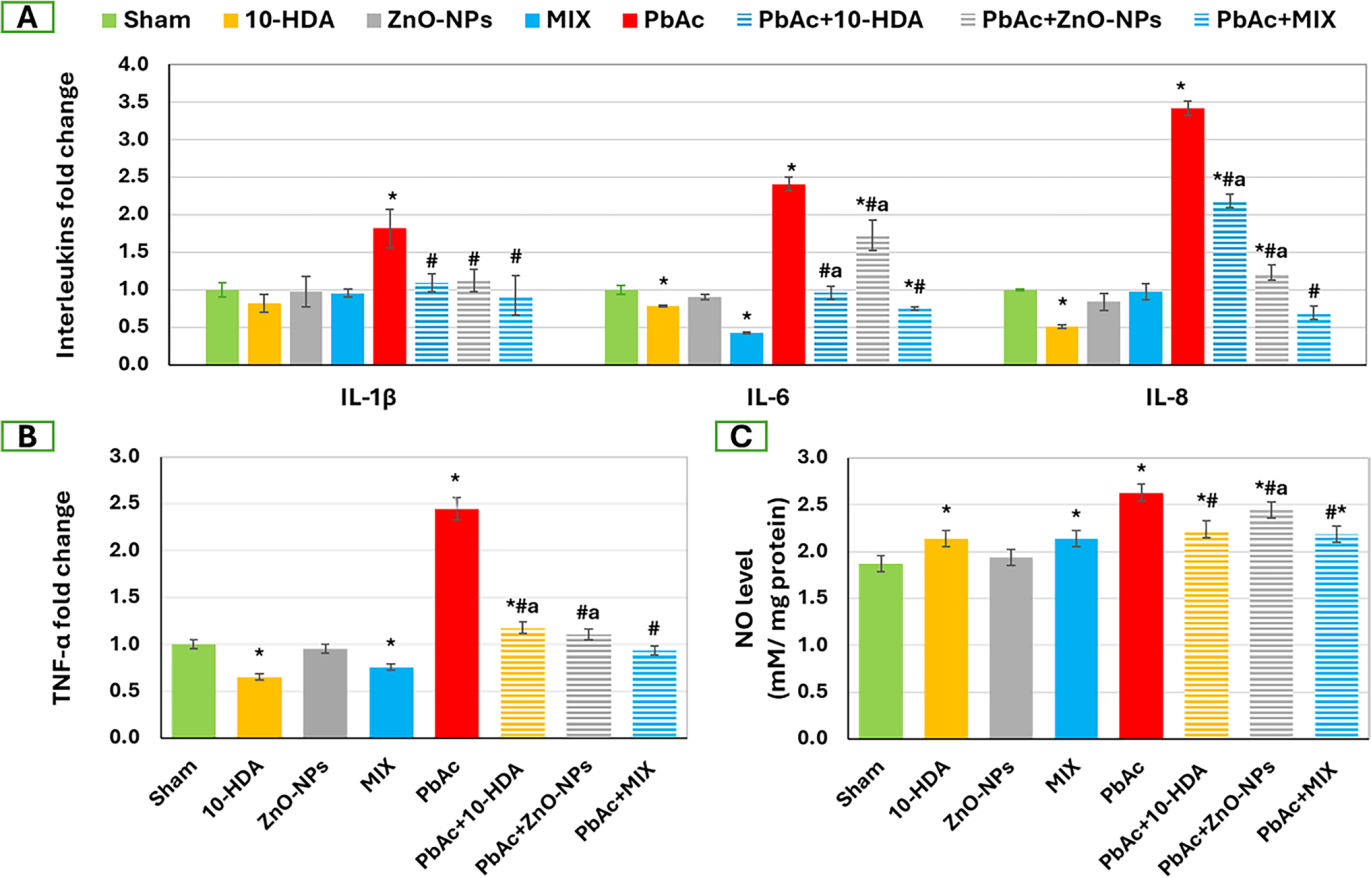
The impact of lead acetate exposure and different treatments on testicular inflammatory markers. (A) Interleukins fold change (IL-1β, IL-6, and IL-8), (B) TNF-α fold change, and (C) NO level. Data is shown as mean ± standard deviation (*n* = 4). *: significant (*p* < 0.05) compared to the sham group, ^#^: significant (*p* < 0.05) compared to the PbAc-induced group, ^a^: significant (*p* < 0.05) compared to the PbAc + MIX group. 10-HDA, 10-hydroxydecanoic acid (5 mg/kg/day),; ZnO-NPs, zinc oxide nanoparticles (5 mg/kg/day); MIX, mixture of 10-hydroxydecanoic acid + Zinc oxide nanoparticles; PbAc, lead acetate (30 mg/kg/day); PbAc + 10-HDA, lead acetate + 10-hydroxydecanoic acid; PbAc + ZnO-NPs, lead acetate + zinc oxide nanoparticles; PbAc + MIX, lead acetate + mixture of 10-hydroxydecanoic acid + zinc oxide nanoparticles

Additionally, as depicted in Fig. 6, the exposure to PbAc resulted in the initiation of testicular inflammation, as evidenced by the statistically significant (*p* < 0.05) increases in the levels of inflammatory markers, namely IL-1β, IL-6, IL-8, TNF-α, and NO, when compared to the control group. In contrast, the administration of 10-HDA and ZnO-NPs, either individually or in combination, to rats induced with PbAc resulted in statistically significant reductions (*p* < 0.05) in these levels when compared to the PbAc-toxified rats that did not receive any treatments. Interestingly, the MIX treatment exhibited a greater efficacy in reducing the markers of inflammation. Most of the control treatments with 10-HDA and/or ZnO-NPs exhibited a significant reduction (*p* < 0.05) in the examined inflammatory markers compared to control rats receiving saline.

### The Impact of Lead Acetate Exposure and Different Treatments on Testicular Apoptotic and Survival Responses Among the Investigated Groups

Rats receiving PbAc demonstrated a significant elevation (*p* < 0.05) in Bax and caspase-3 expression levels compared to the sham group. However, these levels were significantly (*p* < 0.05) reverted in PbAc-treated rats receiving 10-HDA, ZnO-NPs, or MIX compared with PbAc-infertility rats. The controls receiving the treatment protocols showed significant differences (*p* < 0.05) compared to the healthy untreated rats. Regarding the survival marker (Bcl-2), PbAc caused a significant (*p* < 0.05) drop in its expression level compared to the sham rats. Conversely, the administration of 10-HDA and ZnO-NPs individually or in combination resulted in a statistically significant increase (*p* < 0.05) in the expression of Bcl-2 in these rats. In comparison to the sham rats, the control group treated with ZnO-NPs did not exhibit any statistically significant differences in Bcl-2 levels.

**Fig. 7:**
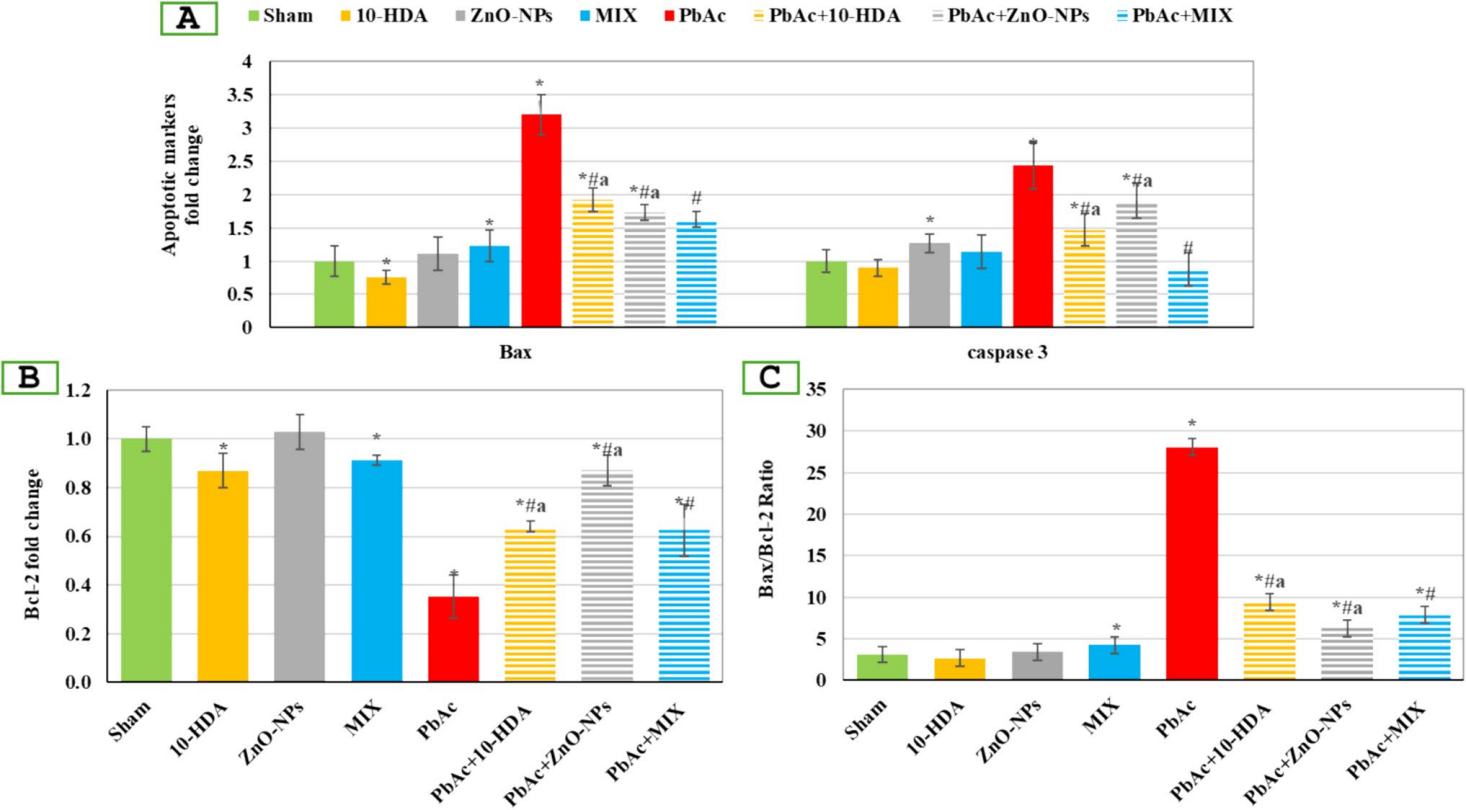
The impact of lead acetate exposure and different treatments on testicular apoptotic and survival markers. (A) Bax and caspase-3 mRNA expression levels, (B) Bcl-2 mRNA expression levels, and (C) Bax/Bcl-2 ratio. Data is shown as mean ± standard deviation (*n* = 4). *: significant (*p* < 0.05) compared to the sham group, ^#^: significant (*p* < 0.05) compared to the PbAc-induced group, ^a^: significant (*p* < 0.05) compared to the PbAc + MIX group. 10-HDA, 10-hydroxydecanoic acid (5 mg/kg/day); ZnO-NPs, zinc oxide nanoparticles (5 mg/kg/day); MIX, mixture of 10-hydroxydecanoic acid + zinc oxide nanoparticles; PbAc, lead acetate (30 mg/kg/day); PbAc + 10-HDA, lead acetate + 10-hydroxydecanoic acid; PbAc + ZnO-NPs, lead acetate + zinc oxide nanoparticles; PbAc + MIX, lead acetate + mixture of 10-hydroxydecanoic acid + zinc oxide nanoparticles

Concerning the Bax/Bcl-2 ratio, the PbAc-treated group showed a significant rise (*p* < 0.05) compared with healthy rats. Remarkably, administration of 10-HDA and/or ZnO-NPs to PbAc-intoxified rats exhibited a significant decline (*p* < 0.05) in the Bax/Bcl-2 ratio compared to PbAc-infertility rats. Only the MIX-control group exhibited a statistically significant increase (*p* < 0.05) in the Bax/Bcl-2 ratio compared to the sham group (Fig. 7).

### Synergistic Properties of 10-HAD and ZnO-NPs Combined Treatment

The combined administration of 10-HDA and ZnO-NPs demonstrated a synergistic effect (CI < 1) against lead acetate–induced oxidative stress, inflammation, and apoptosis actions in rats’ testicular tissues (Table 4).

**Table 4:** Combination index (CI) of 10-HDA and ZnO-NPs mixture on the measured parameters

Parameters		CI	Effect
Seminal parameters and hormone levels			
Sperm count (10^6^/ml)		0.550 ± 0.033	Synergistic
Morphology index (%)		0.505 ± 0.022	Synergistic
Motility type (%)	Rapid	0.526 ± 0.013	Synergistic
	Non-progressive	0.460 ± 0.015	Synergistic
Motility (%)	1st h	0.575 ± 0.028	Synergistic
	2nd h	0.616 ± 0.037	Synergistic
	3rd h	0.779 ± 0.034	Synergistic
Seminal fructose level (mg/g tissue)		0.466 ± 0.022	Synergistic
FSH (µU/ml)		0.507 ± 0.058	Synergistic
LH (µU/ml)		0.671 ± 0.054	Synergistic
Testosterone (ng/dl)		0.700 ± 0.098	Synergistic
Testicular lead content (ppm)		0.369 ± 0.041	Synergistic
Oxidative stress markers			
SOD (U/mg protein)		0.543 ± 0.011	Synergistic
GST (U/min/mg protein)		0.521 ± 0.030	Synergistic
GSH (mM/g tissue)		0.534 ± 0.083	Synergistic
NO (mM/mg protein)		0.496 ± 0.205	Synergistic
MDA (mM/mg protein)		0.373 ± 0.047	Synergistic
Nrf2 (protein expression level)		0.532 ± 0.015	Synergistic
HO-1 (protein expression level)		0.624 ± 0.027	Synergistic
Inflammatory markers			
p-IKK (protein expression level)		0.270 ± 0.071	Synergistic
IL-1β (mRNA fold change)		0.488 ± 0.057	Synergistic
IL-6 (mRNA fold change)		0.318 ± 0.039	Synergistic
IL-8 (mRNA fold change)		0.239 ± 0.081	Synergistic
TNF-α (mRNA fold change)		0.474 ± 0.042	Synergistic
Apoptotic markers			
Caspase-3 (mRNA fold change)		0.304 ± 0.059	Synergistic
Bcl-2 (mRNA fold change)		0.498 ± 0.058	Synergistic
Bax (mRNA fold change)		0.513 ± 0.025	Synergistic

CI value of < 1 indicates synergistic effect; > 1 indicates antagonistic effect; = 1 indicates additive effect. Values are means ± SD

### The Impact of Lead Acetate Exposure and Different Treatments on Testicular Histoarchitecture

**Fig. 8:**
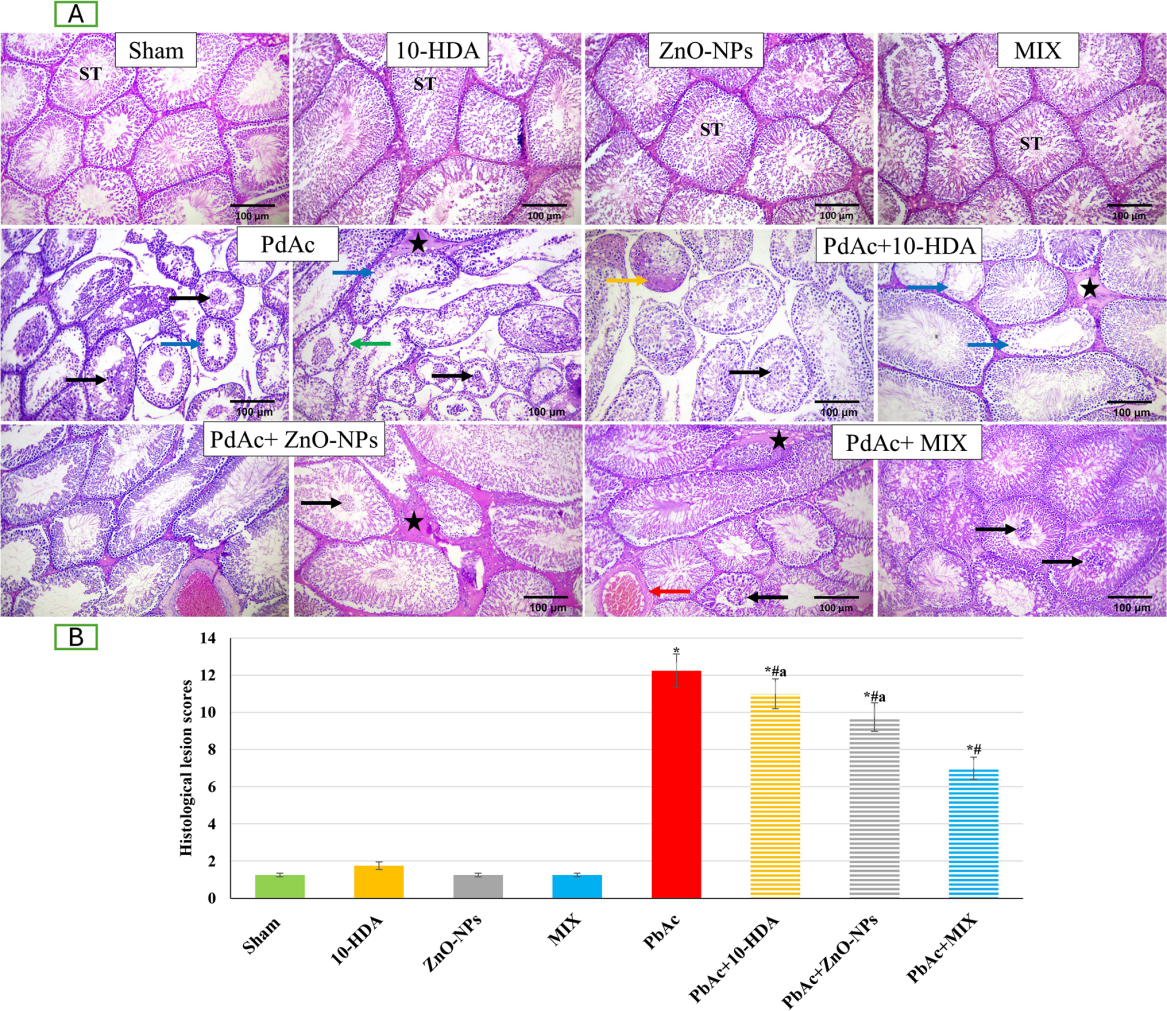
Representative photomicrographs demonstrating the histopathological changes in the rat testicular tissues of different groups. (A) H&E stained sections (× 100). Normal seminiferous tubules (ST), a typical seminiferous tubules with deformed spermatogenesis and buckled tubular membrane (green arrow), sloughed deteriorated germinal epithelial cells (black arrow), diminution of the germinal epithelium of tubules (blue arrow) with necrosis and hyalinization of the luminal contents (orange arrow), congestion of interstitial vessel (red arrow) and interstitial edema (star) were detected. (B) Histological lesion score assessment across various groups. Data is shown as mean ± standard deviation (*n* = 10). *p*-values were assessed by comparing them with those of the sham group. *: significant (*p* < 0.05) compared to the sham group, ^#^: significant (*p* < 0.05) compared to the PbAc-induced group, ^a^: significant (*p* < 0.05) compared to the PbAc + MIX group. 10-HDA, 10-hydroxydecanoic acid (5 mg/kg/day); ZnO-NPs, zinc oxide nanoparticles (5 mg/kg/day); MIX, mixture of 10-hydroxydecanoic acid + zinc oxide nanoparticles; PbAc, lead acetate (30 mg/kg/day); PbAc + 10-HDA, lead acetate + 10-hydroxydecanoic acid; PbAc + ZnO-NPs, lead acetate + zinc oxide nanoparticles; PbAc + MIX, lead acetate + mixture of 10-hydroxydecanoic acid + zinc oxide nanoparticles

Figure 8 demonstrates the testicular tissues of sham, 10-HDA, ZnO-NPs, and MIX groups. The examined samples displayed normal histological and architectural features of interstitial tissues, seminiferous tubules, Sertoli cells, Leydig cells, and spermatogenic cells. Conversely, spermatogenic cells of testicular samples from rats administering PbAc revealed anomalous arrangement, vacuolization, and degeneration. Additionally, seminiferous tubules exhibited a necrotic epithelial cell lining and a detached basement membrane. When examining the central regions of the tubular laminae, tissue samples obtained from rats induced with PbAc exhibited the presence of cellular debris, necrosis of spermatocytes, and eosinophilic proteinaceous materials. Furthermore, there is a marked decline in Sertoli cell count and expanded intertubular tissues with scarce Leydig cells spreading, along with intertubular blood vessel congestion and interstitial edema. Conversely, the earlier deteriorations and evident amendments in the testicular histological and architectural structures were ameliorated by the treatment of the PbAc-induced testicular toxicity with either or both 10-HDA and ZnO-NPs. Nevertheless, their efficacy was comparable to that of the sham limitations. The group treated with PbAc + MIX exhibited the most significant ameliorating effect.

## Discussion

Lead is a heavy metal that is considered non-essential for living organisms. It possesses the ability to induce a wide range of physiological and biochemical alterations, leading to renal failure, neurological dysfunctions, impaired fertility, and eventually death [67]. Lead has the potential to cause male infertility through various mechanisms, including its interference with the production of antioxidant enzymes. It increases free radicals production, impairs signaling pathways, provokes apoptosis, increases inflammation, causes cellular dysfunction, and impedes endocrine function through the hypothalamus-pituitary-testes axis [68, 69]. Additionally, lead is well known for its low elimination rate, thereby promoting its accumulation within the human body [70]. Lead accumulation in reproductive tissues can compromise the integrity of the blood-testis barrier, which is predominantly reinforced by the tight junctions present in Sertoli cells. Consequently, the compromised blood-testis barrier facilitates lead entry into the seminiferous tubules, amplifying lead toxicity [71]. In the current study, a substantial elevation in the testicular lead content was observed, accompanied by a depleted sperm count, morphology index, and motility, in rats exposed to PbAc. Furthermore, PbAc exposure resulted in histopathological alterations in testicular tissues represented by epithelium vacuolization, diminished diameter of seminiferous tubules, elevated apoptosis of germ cells, impaired spermatogenesis, and substantial maturation arrest [72, 73]. These findings provide further support for previous studies indicating that the accumulation of lead in testicular tissues and the disruption of lead homeostasis in male reproductive organs can have detrimental effects on the integrity of the blood-testis barrier, sperm production and morphology, sperm count, sperm motility, and the rate of DNA damage in sperm cells [74–78].

The current study aimed to determine the curative effect of 10-HDA and its combination with ZnO-NPs on male infertility, testicular antioxidants, reproductive hormones, pro-inflammatory and pro-apoptotic gene expression, and histopathology. This study hypothesized that the 10-HDA and ZnO-NPs could have beneficial effects on the male rat reproductive system compared to their monotherapy. ZnO-NPs possess distinctive physical and chemical characteristics that facilitate their interaction with cellular macromolecules and elicit a range of medicinal effects through expressing antioxidants, antibacterial, anticancer, and anti-inflammatory potentials [79–82]. Goma [49] demonstrated that ZnO-NPs at a low dose of 5 mg/kg can have a positive impact on male fertility. However, high doses (50, 150, 300, and 350 mg/kg) may function as testicular toxicants in mice [49, 83]. 10-HDA has a stable structure in RJ that is free of double bonds [28]. Several studies reviewed the favorable functions of RJ on male fertility [23, 84, 85]. Oral administration of RJ (100 mg/kg) in diet before sexual maturity improved semen quality and sexual behavior of male rabbits [84]. Furthermore, the antioxidant properties of RJ facilitate the mitigation of cellular toxicity in testicular tissue and protect the integrity of the testicular structure against the detrimental impacts of doxorubicin [86] and diabetic oxidative stress [85], hence preserving male fertility. The present study demonstrates that the administration of 10-HDA and/or ZnO-NPs resulted in a reduction in testicular lead content, indicating the chelating activity of our treatment and subsequent lead clearance from the testes. Remarkably, the combined treatment exhibited a synergistic activity by effectively lowering lead content in testicular tissues. Additionally, the treatments significantly improved sperm count, morphology index, and motility and were able to improve the architecture of testicular tissues. In agreement with our findings, Afifi [87] illustrated that ZnO-NPs (10 mg/kg), either alone or in combination with insulin, can increase sperm count and motility and shield testicular tissue from oxidative stress in diabetic rats. Moreover, the co-administration of green ZnO-NPs showed adequate protective effects against the induced testicular damage [50].

Sperm functionality is adversely affected when ROS levels surpass the physiological threshold, which is necessary for normal sperm functioning [3, 88]. The toxicity of lead is associated with an increase in oxidative stress, resulting in harm to DNA, proteins, and lipids. These detrimental effects can accelerate the deterioration of both the functionality and structure of the testes, ultimately leading to cell death and involvement in various diseases [78]. Lead can trigger oxidative stress by two routes: the amplified production of ROS and the impact of lead on the antioxidant defense system [89]. The excessive production of ROS is amalgamated with various male fertility matters, for example, varicocele and leukocytopenia [3, 88, 90]. In the present study, rats receiving PbAc demonstrated a decline in the activity of SOD and GST, as well as the content of the antioxidant GSH, indicating a compromised antioxidant defensive system. These outcomes align with the preceding findings [91, 92]. Conversely, 10-HDA and ZnO-NPs therapies could alleviate testicular tissue oxidative stress by increasing SOD and GST activities and GSH content along with reduced MDA levels. The antioxidant properties of these treatments may be responsible for these recoveries. In two microglial models, 10-HDA was reported to suppress the production of ROS, which was induced by lipopolysaccharide (LPS), in a dose-dependent manner [22]. Additionally, RJ (100 mg/kg/day, for 35 days) was reported to effectively protect the testis against nicotine-induced testicular damage through upregulation of the antioxidant activity, prevention of mitochondria-dependent apoptosis, and the improvement of proliferating activity [93]. Zinc is a cofactor for approximately 300 enzymes that play a variety of significant activities, primarily those that are engaged in the antioxidant defense process. Zinc serves as a sulfhydryl group protector, preventing it from adhering to catalytic sites, and is essential for the activation of antioxidant enzymes and proteins, such as catalase, SOD, and glutathione [94, 95]. In ZnO-NPs treated diabetic rats, Afifi [87] demonstrated increased SOD, GST, glutathione peroxidase, glutathione reductase, and catalase activity and mRNA expression. Moreover, testicular tissue MDA decreased, while GSH increased. Additionally, Majd [50] illustrated that the co-administration of green ZnO-NPs (5 mg/kg) restored the suppressive effects of cisplatin-induced reproduction toxicity on the activities of the antioxidant enzymes (SOD, glutathione peroxidase, and catalase). Therefore, low doses of 10-HDA and ZnO-NPs improved the antioxidant status of the testicular tissue, reduced free radical levels, and shielded the integrity of the cell membrane from the damaging effects of oxidative stress.

Additionally, Nrf2 and HO-1 constitute a vital pathway that protects against various environmental and internal stressors [96]. The protein Nrf2, which plays a crucial role in regulating antioxidant proteins, is generally rendered inactive through its interaction with the cytoplasmic complex. However, upon cellular damage, Nrf2 gets translocated into the nucleus to stimulate the production of a variety of phase II detoxification and antioxidant enzymes, such as glutathione reductase, NAD(P)H quinone dehydrogenase-1 (NQO-1), and HO-1. These enzymes mitigate oxidative stress in cells and tissues [97, 98]. This study demonstrated reduced protein levels of Nrf2 and HO-1 in the testes of the PbAc-intoxicated group. These results align with previous findings [99, 100]. Alternatively, 10-HDA and ZnO-NPs treatments restored Nrf2 and HO-1 protein levels, reflecting these treatments’ ability to reestablish the antioxidant defense system through reactivating the Nrf2/HO-1 signaling pathway. Consistent with these findings, Almeer [101] deduced that RJ administration minimized the Cd-induced testicular toxicity and oxidative stress through the upregulation of Nrf2 and HO-1. The study of Awadalla [98] indicated that intraperitoneal injection of ZnO-NPs (5 mg/kg, 3 times/week, for 8 weeks) up-regulated antioxidant genes (Nrf2 and HO-1) in the induced-chronic kidney disease rats.

Mitochondrial dysfunction is associated with disrupted steroidogenesis and spermatogenesis. Lead could induce mitochondrial impairment by triggering oxidative stress, mitochondrial membrane depolarization, and permeabilization while inhibiting enzymes involved in energy metabolism [102]. Moreover, the significant amount of polyunsaturated fatty acids (PUFA) and the abundance of mitochondria in spermatozoa render them more susceptible to oxidative stress [103]. ROS can impair sperm viability, morphology, motility, and capacity by inducing oxidative DNA strand breaks, mitochondrial dysfunction, and PUFA peroxidation. These detrimental effects can cause metabolic dysfunction of Sertoli cells, thus impairing spermatogenesis [104, 105]. The current investigation revealed that exposure to PbAc resulted in a notable decrease in sperm count, morphology index, and motility characteristics. Furthermore, PbAc induced an increase in testicular MDA, which is the main reaction product of lipid peroxidation. These changes are supported by previous investigations [16, 106–108]. Treating rats with 10-HDA and/or ZnO-NPs produced a profound enhancement in sperm count, morphology index, motility, and decreased MDA levels. RJ’s improved activity on sexual behavior and semen quality was previously reported as contributing to its antioxidant properties [84–86, 109]. ZnO-NPs represent a valuable and safe additive material that is effective at reducing peroxidative damage during sperm cryopreservation and improving semen quality by acting as antioxidants [82, 110, 111]. Interestingly, Majd [50] reported that the co-administration of green ZnO-NPs to cisplatin-induced reproduction toxicity significantly elevated sperm count and progressive motility, while this increasing effect was not observed in ZnO bulk and chemical ZnO-NPs. This finding is compatible with the synergetic effect of the current combination of ZnO-NPs with 10-HAD. The preventive and ameliorative effects of 10-HAD and ZnO-NPs on sperm parameters can be attributed to their antioxidant and free radical scavenging ability.

Seminal fructose is the key energy source for spermatozoa. It serves as a marker of seminal vesicle function [112]. Lead can lower seminal fructose, which can be related to the ability of lead to replace metal cofactors of different enzymes involved in the biosynthesis of fructose [105, 113]. In contrast, the application of 10-HDA and/or ZnO-NPs agents effectively reversed the decrease in seminal fructose levels resulting from exposure to PbAc. This finding could potentially be elucidated by the antioxidant capacity of our therapeutic interventions, as prior research has established a correlation between reduced seminal fructose levels and the occurrence of oxidative stress [105, 112, 114]. Furthermore, the administered remedies possess chelating properties that effectively reduce the levels of lead in the testes, thereby facilitating the restoration of enzymatic activity involved in fructose synthesis.

Another mechanism that contributes to male infertility is testicular inflammation [115]. Oxidative stress is immensely implicated in the development of inflammation. This result can be explained by the ability of reactive oxygen/nitrogen species to activate intracellular signaling pathways that encourage the activation of pro-inflammatory responses [116]. Furthermore, inflammatory mediators can activate and recruit immune cells to the inflammatory site. These cells induce an excessive release of reactive species, intensifying oxidative stress [117]. Besides, NO produced by iNOS during oxidative stress can aggravate inflammation by amplifying NF-κB signaling [118–120]. NF-κB signaling pathway controls the expression of pro-inflammatory cytokines and chemokines. In the cytoplasm, IkB sequesters NF-κB, thereby inhibiting its activity. TNF-α activates NF-κB signaling through phosphorylating IKK, which sequentially phosphorylates IkB, causing its degradation and releasing NF-κB to initiate target gene transcription [121]. In this investigation, PbAc exposure caused an increase in testicular NO content, escorted by a rise in the expression of p-IKK, IL-1β, IL-6, IL-8, and TNF-α. This finding is consistent with previous studies reporting the stimulation of the NF-κB pathway accompanied by an increase in inflammatory cytokines in testicular tissue following lead administration [102, 122–126].

In contrast, previous studies reported the anti-inflammatory activity of 10-HDA by hindering the NF-κB pathway and the expression of pro-inflammatory cytokines [22, 118, 127]. The study of You [22] revealed that the increased iNOS, IL-1β, IL-6, and TNF-α levels were suppressed by the 10-HDA pretreatment associated with a concentration-dependent decrease in NO levels compared to LPS-induced inflammation. Furthermore, the anti-inflammatory properties of ZnO-NPs were demonstrated through the reduction of NF-κB signaling and the subsequent decrease in pro-inflammatory cytokines. Moreover, the utilization of ZnO-NPs has been shown to inhibit the expression of iNOS, thereby reducing inflammation [128, 129]. Goma [49] and Awadalla [98] reported down-regulated inflammatory genes (IL-6 and TNF-α) in ZnO-NP-treated rats. In consistence, the present treatments involving 10-HDA and/or ZnO-NPs reduced the levels of p-IKK and the pro-inflammatory mediators. Therefore, we postulate that the inhibition of the NF-κB pathway may be involved in the mechanism by which 10-HDA and ZnO-NPs exert their anti-inflammatory effects.

The hypothalamic-pituitary–testicular axis can be negatively impacted by lead-induced oxidative stress and inflammation, which can lead to the inhibition of testosterone production and spermatogenesis through the induction of gonadotropin-inhibitory hormone expression [104, 130]. Moreover, lead can hamper the hormonal feedback pathways in the reproductive hormonal axis by dysregulating LH and FSH production. Oxidative stress and inflammation are correlated with elevated prolactin, exerting a negative feedback loop on LH, FSH, and testosterone production, reducing spermatogenesis [131]. Additionally, lead can directly hinder testicular steroidogenesis by impeding Leydig cell differentiation, which is responsible for the production of testosterone [132]. Consistent with previous findings, PbAc-exposed rats displayed a significant decline in the blood sex hormone levels (testosterone, LH, and FSH) [108, 133, 134]. 10-HDA and/or ZnO-NPs administration revealed a notable rise in testosterone, FSH, and LH levels compared to PbAc-exposed rats. The observed decline in activity may be attributed to oxidative stress induced by a reduction in the efficacy of antioxidant species. Hence, the observed enhancement could potentially be attributed to the potent antioxidant characteristics exhibited by these compounds. Furthermore, the administration of RJ not only mitigates reproductive tract impairment resulting from various detrimental stressors but also enhances the production of male hormones, thereby leading to improvements in sperm count, motility, and morphology [23, 85]. Moreover, there are numerous important biological hormone interactions with zinc. Zinc improved the passivity of organ reactivity and receptor locations and had a role in hormone synthesis, storage, and discharge. Furthermore, it is worth noting that zinc oxide (ZnO) plays a vital role in the synthesis of various sex hormones, including testosterone. The utilization of ZnO as a zinc source may contribute to raising testosterone concentrations [49, 135]. Additionally, Mozaffari [136] stated that ZnO-NPs caused a substantial rise in FSH and testosterone levels. The two intracellular mechanisms by which ZnO-NPs increase LH and FSH are described. One factor contributing to the observed phenomenon is the increased levels of zinc in the pituitary gland. These elevated zinc levels disrupt the release of dopamine from the pituitary gland, consequently reducing the indirect inhibitory effect of dopamine on the gonadotropin-releasing hormone (GnRH). The second mechanism involved the direct inhibition of the gamma-aminobutyric acid (GABA) nerve system by ZnO-NPs [136].

Furthermore, oxidative stress can trigger apoptosis by disrupting the permeability of the mitochondrial membrane. This leads to the liberation of cytochrome c into the cytosol, activating the caspase cascade and promoting apoptotic cell death [137, 138]. One of the anti-apoptotic proto-oncogenes is Bcl-2. Bcl-2 maintains the integrity of the mitochondrial membrane during intrinsic apoptosis. Therefore, the reduction in Bcl-2 expression/stability leads to releasing of cytochrome c through the mitochondrial membrane, resulting in caspase-dependent apoptosis [93]. In line with our findings, PbAc exposure was found to induce apoptosis by enhancing the transcription of the pro-apoptotic Bax and caspase-3 while decreasing the transcription of the anti-apoptotic Bcl-2 in the testicles [125, 139, 140]. The present investigation demonstrated that low doses of 10-HDA and/or ZnO-NPs exhibited anti-apoptotic properties, as indicated by the restoration of the Bax/Bcl-2 ratio and a reduction in the expression level of caspase-3. Similarly, these studies revealed that RJ can modulate oxidative stress, inflammation, and apoptosis and prevent testicular [93], hepato-, and renal [141, 142] toxicity in rats. ZnO-NP anti-apoptotic action was also illustrated [49]. These positive effects can be attributed to the antioxidant and anti-inflammatory properties of our treatments, which enhance their effectiveness through a synergistic combination [143]. Conversely, a single intraperitoneal injection of high-dose ZnO-NPs (700 mg/kg) [49] and daily oral administration of ZnO-NPs (50 mg/kg) for 50 consecutive days [83] have apoptotic effects on spermatogenic cells and resulted in reduced sperm motility and elevated sperm abnormalities. Therefore, it is necessary to ensure sufficient intake of ZnO-NPs in order to maintain the optimal functionality of the testicles.

## Conclusion

**Fig. 9:**
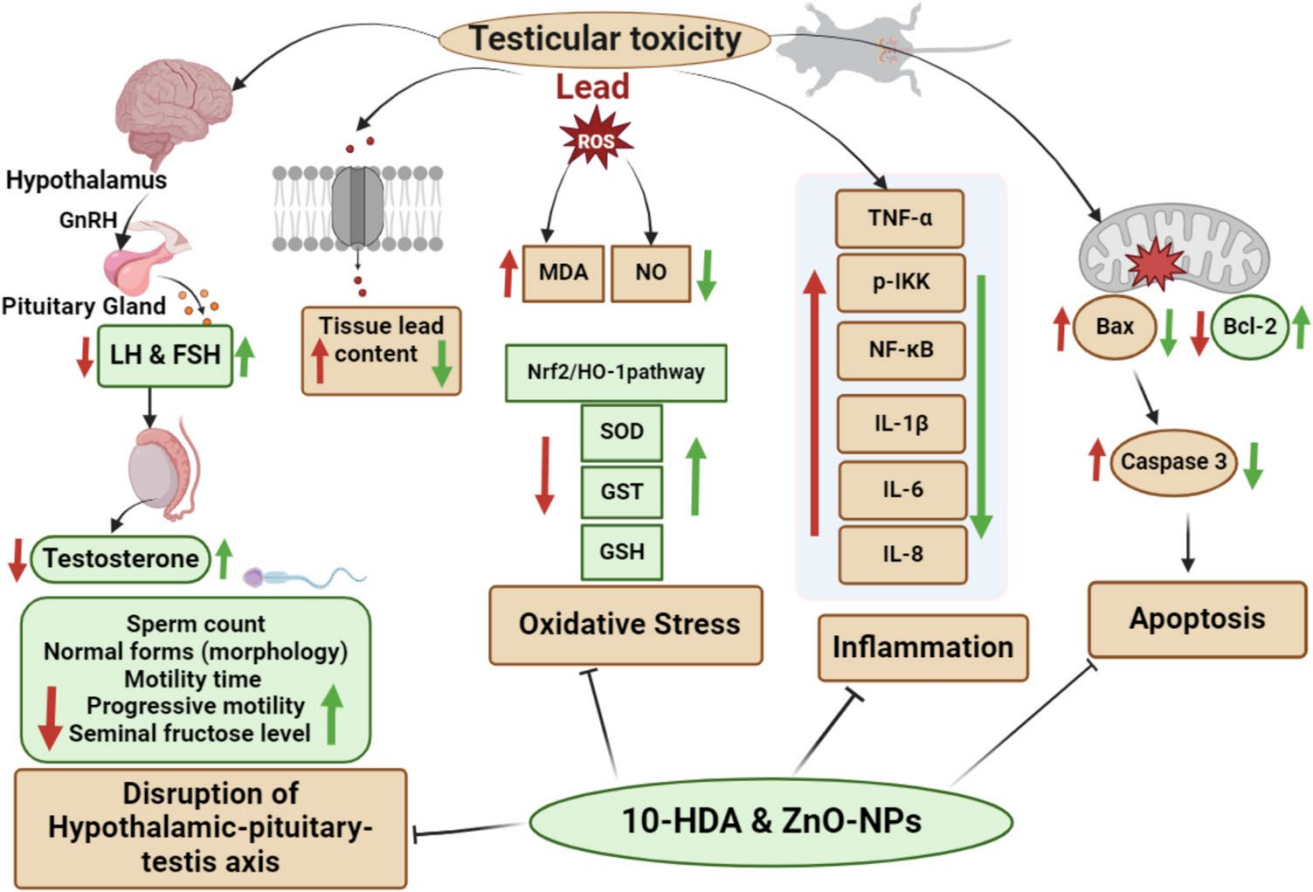
Testicula toxicity as a result of chronic PbAc exposure. PbAc exposure triggers ROS production that disrupts the hypothalamic-pituitary-testis (HPT) axis, resulting in decreased levels of LH, FSH, and testosterone. In addition, it diminishes semen quality by altering sperm morphology and reducing sperm count, motility, progressive motility, and seminal fructose levels. Furthermore, PbAc exposure enhances lead accumulation in testicular tissue and promotes oxidative stress by increasing NO production and MDA levels and inhibiting the Nrf2/HO-1 pathway, leading to a reduction in antioxidant enzymes (SOD, GST, GSH). PbAc exposure also triggers inflammatory signaling by activating TNF-α, p-IKK, NF-κB, and ILs and initiates apoptotic action through the disruption of the caspase-3/Bax/Bcl-2 signaling pathway. The combined therapy of 10-HDA and ZnO-NPs effectively counteract these harmful effects. It restores the HPT axis function, reduces oxidative stress, inhibits inflammation, and prevents apoptosis, thereby protecting testicular function and enhancing fertility

PbAc exposure induces an increase in oxidative stress, resulting in inflammation, testicular damage, and cell death through NF-κB activation and Nrf2/HO-1 inhibition in testicular tissues (Fig. 9). The current study was the first to investigate the impact of 10-HDA and its combination with ZnO-NPs on male reproductive function, hormone levels, antioxidant levels, anti-inflammatory gene expression, and testicular histopathology. The results revealed that 10-HDA and ZnO-NPs at 5 mg/kg improved the semen characteristics, reproductive hormones, and lead clearance, increased testicular antioxidant activity, and the mRNA expression of the different anti-inflammatory and anti-apoptotic genes. In conclusion, the co-administration of ZnO-NPs with 10-HDA therapy demonstrated the most beneficial effect on male reproductive function compared to the use of these remedies independently. Individuals who are regularly exposed to lead will greatly benefit from this study.

Limitations: Further studies on the mechanisms of lead detoxification by 10-HDA and ZnO-NPs are necessary in order to elucidate the precise mechanism underlying the testicular ameliorative efficacy of these treatments. Moreover, an in silico molecular modeling study is needed to gain a comprehensive understanding of the interaction between the used remedies and various proteins. This study aims to assess the pharmacokinetics, pharmacodynamics, and tissue distribution of 10-HAD and ZnO-NPs, taking into consideration their potential future applications. However, animals were exposed to the target chemicals for a brief period in this investigation. It is possible that the prolonged use of these substances could be hazardous. Therefore, it is necessary to investigate the potential toxicity of these chemicals due to long-term use. Further research in this area may strengthen the case for a combination medicine with greater efficacy and fewer adverse effects.

## Data Availability

The authors declare that the data supporting the findings of this study are available within the manuscript.
