# Correction: 10-Hydroxy Decanoic Acid and Zinc Oxide Nanoparticles Retrieve Nrf2/HO-1 and Caspase-3/Bax/Bcl-2 Signaling in Lead-Induced Testicular Toxicity

**DOI:** 10.1007/s12011-024-04491-z

**Published:** 2024-12-20

**Authors:** Adham M. Maher, Ghidaa A. Elsanosy, Doaa A. Ghareeb, Samar S. Elblehi, Samar R. Saleh

**Affiliations:** 1 Bio-Screening and Preclinical Trial Lab, Department of Biochemistry, Faculty of Science, Alexandria University, Alexandria, 21511 Egypt; 2 Pharmaceutical and Fermentation Industries Development Centre (PFIDC), The City of Scientific Research and Technological Applications (SRTA-City), Alexandria, Borg Al‑Arab Egypt; 3 Research Projects Unit, Pharos University, Alexandria, Egypt; 4 Department of Pathology, Faculty of Veterinary Medicine, Alexandria University, Alexandria, 21944 Egypt

**Correction to: Biological Trace Element Resear**

10.1007/s12011-024-04374-3

The published version of this article unfortunately contained a mistake.

In Fig. 6b of this article PbAc has a wrong a and * at the last 2 groups; the Fig. 6b should have appeared as shown below.
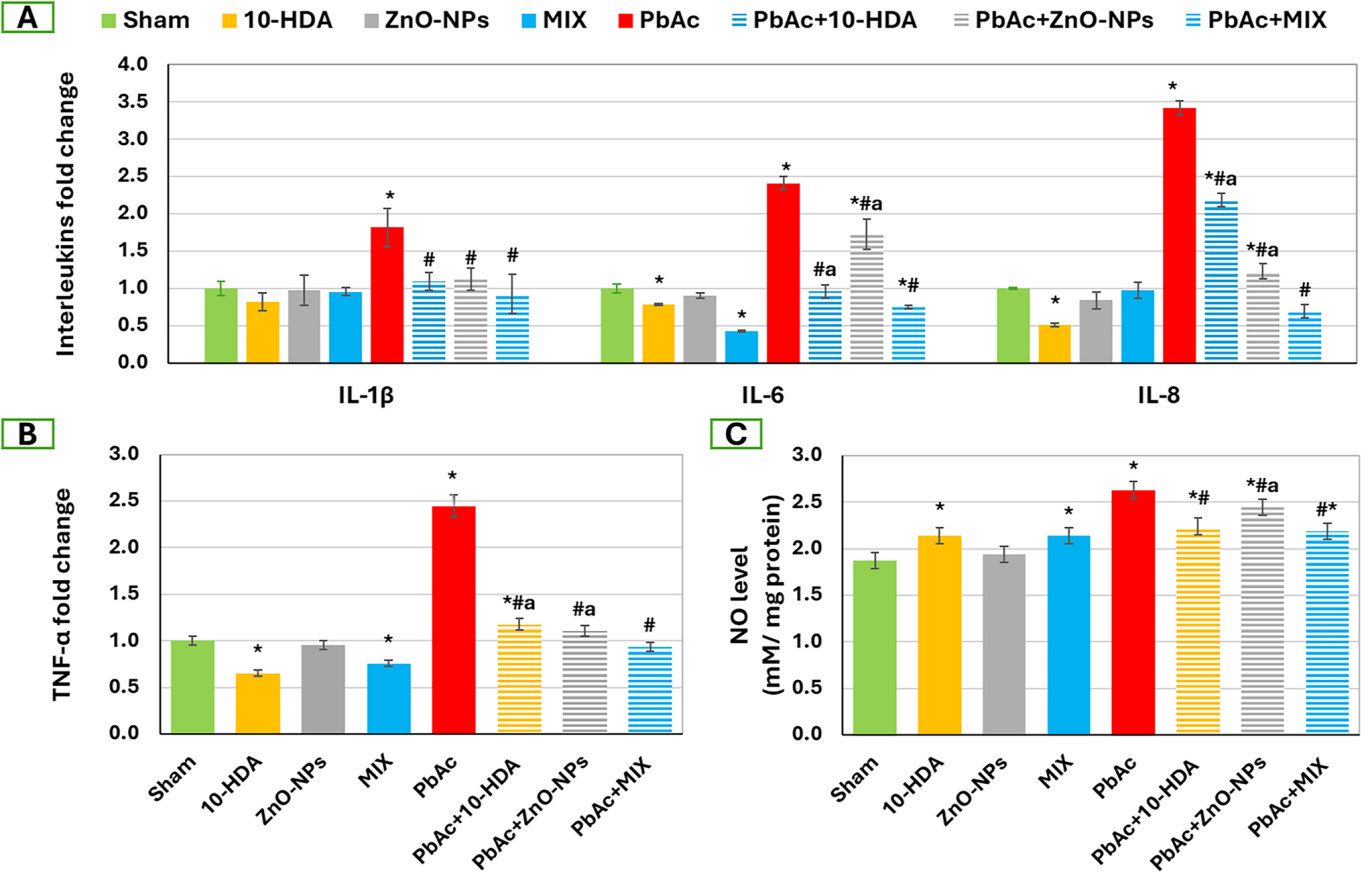


The original article has been corrected.

